# Alcohol Health Warning Labels: A Rapid Review with Action Recommendations

**DOI:** 10.3390/ijerph191811676

**Published:** 2022-09-16

**Authors:** Norman Giesbrecht, Emilene Reisdorfer, Isabelle Rios

**Affiliations:** 1 Centre for Addiction and Mental Health, 33 Ursulla Franklin St., Toronto, ON M5S 2S1, Canada; 2 Dalla Lana School of Public Health, University of Toronto, 155 College St., Toronto, ON M5T 3M7, Canada; 3 Faculty of Nursing, MacEwan University, Edmonton, AB T5J 4S2, Canada; 4 Independent Researcher, USA

**Keywords:** alcohol, health warning labels, knowledge, awareness, behavior, pregnancy, cancer, design, message, policy recommendations

## Abstract

A rapid review of research on health warning labels located on alcohol containers (AWLs) was conducted. Using five search engines (Embase, Medline, Pubmed, Scopus, Psyinfo), 2975 non-duplicate citations were identified between the inception date of the search engine and April 2021. Of those, 382 articles were examined and retrieved. We selected 122 research papers for analysis and narrative information extraction, focusing on population foci, study design, and main outcomes. Research included public opinion studies, surveys of post-AWL implementation, on-line and in-person experiments and real-world quasi-experiments. Many studies focused on the effects of the 1989 United States Alcoholic Beverage Labeling Act on perceptions, intentions and behavior. Others focused on Australia, Canada, the United Kingdom, England or Scotland, Italy and France. There was substantial variation in the design of the studies, ranging from small-scale focus groups to on-line surveys with large samples. Over time, evidence has been emerging on label design components, such as large size, combination of text and image, and specific health messaging, that is likely to have some desired impact on knowledge, awareness of risk and even the drinking behavior of those who see the AWLs. This body of evidence provides guidance to policy-makers, and national and regional authorities, and recommendations are offered for discussion and consideration.

## 1. Introduction

Effective public health policy responses to alcohol-related harm consist of a number of components [1], including, but not limited to, controls on physical availability, precautionary pricing and taxation, controls on drinking and driving, marketing controls and screening, brief intervention and treatment. Conveying information on the harms and risks of alcohol consumption is an essential component of the overall alcohol strategy of a jurisdiction [2,3]. This might be done at the population level through counter-advertising or other media-based strategies. At the community level, seminars, town halls, and other events can convey information about harms from alcohol. At the individual level, this information can be provided via advice offered by an educator, physician, nurse or other health care worker to the drinker or potential drinker. Individuals may also receive information on the harms and risks of alcohol consumption via warning labels found on alcohol containers. A warning label is a well-established and frequently used method for conveying information about risks associated with various products. Warning labels placed on alcohol containers have the potential to be seen by consumers, and likely more often by those who drink more frequently. There are several justifications for having warning labels on alcohol containers:

- Alcohol is associated with a number of social, chronic and acute risks and potential harms and the consumer has the right to know what the potential impacts might be to them, or others, such as a victim of drinking and driving [4].
- The consumer has a right to know what they are drinking or being served, and the potential risks involved [5].
- A warning label is a well-established and frequently used method for conveying information about risks associated with various other products [6].
- Warning labels are likely to be seen more often by frequent consumers, so it is a cost-effective way of conveying basic information about certain alcohol-related risks [7].
- Alcohol warning labels (AWL) potentially offer support for other alcohol strategies and interventions, such as precautionary pricing of alcohol, controls on access to alcohol, restrictions on alcohol marketing, and promotion of low-risk drinking guidelines, by offering an indirect rationale for these policies and reinforcing the effectiveness of these other measures [8].
- While labels may not necessarily change drinking behavior, they may provide increased awareness that alcohol is no ordinary commodity and thus deserving of special attention and concern [9].

A fact sheet developed by the Pan American Health Organization (2022) [10] indicated that, globally, 43 countries use AWLs. As of 2019, there were 14 countries in the Americas with mandated labels and 14 with voluntary labels [11]. Alcohol warning labels (AWLs) are mandated in the ten countries of the Commonwealth of Independent States [12].

Many of the AWLs currently in use, such as a symbol about not drinking and driving, or a “please drink responsibly” phrase, can be hypothesized to be of very low potency to change knowledge, intentions, or behavior. Many AWLs are introduced without plans to assess their impact on knowledge, perceptions of risk or drinking behavior. 

In the course of our research, we encountered a number of reviews with varying scope, aims and quality [12,13,14,15,16,17,18,19,20,21,22,23,24,25,26,27,28,29,30,31,32,33,34,35]. Although the recent scoping review by Kokole et al. [36] was published after the cut-off date for our search, we highlight some key points from it.

We identified 25 reviews, with the number of original studies cited in these reviews ranging from 4 [31] to 71 [28]. They were mainly narrative reviews, with systematic reviews by Schloes-Balog [16] and Hassan and Shiu [37]. Thomas et al. [19] conducted a brief review and Dimova and Mitchell [28] a rapid review. Knai et al. [38] reported on a systematic review of reviews and Robinson et al. [27] conducted a rapid systematic review and metanalysis. A recent report from the WHO Regional Office for Europe on current alcohol-labelling practices in that region [39] noted that 28% of member states had some legislation on certain aspects of health information labelling, with a main focus being pregnancy or underage drinking. 

Some reviews [31,33,40] tended to conclude that there was not convincing evidence of changes in alcohol-related behavior as a result of an AWL. In contrast, Stockwell [35] highlights that the US AWLs raised awareness, particularly among higher risk drinkers, and appeared to stimulate discussions about alcohol issues.

Furthermore, Wilkinson and Room [14] point to AWLs having the potential to change the conversation about alcohol—that it should it be perceived as not an ordinary commodity and thus one requiring special treatment. Thomas et al. [19] noted that AWLs have been shown to stimulate conversations and may play a role in shifting social norms to reduce risks.

Robinson et al. [27] called for real-world experiments to address the concerns that they raise about the low methodological quality found in the studies that they reviewed. Dimova and Mitchell [28] conclude that well-designed AWLs can positively influence consumers’ attention, comprehension, recall, judgement and behavioral compliance. A comparable general conclusion is drawn by Kokole et al. [36], namely, that real-world research has shown that AWLs that are designed to be visible and have novel and specific information can be effective in raising awareness of alcohol-related risks.

Our rapid review complements and builds on this work; it is wider in scope in many cases, and covers a longer time period than most other reviews. Furthermore, our review provides a historical overview of the development of alcohol warning labels and policies related to its application. It also explores how the research on the topic evolved over the years and makes suggestions and recommendations for future action. 

## 2. Materials and Methods

A rapid review of the literature was conducted with the aim to assess the scientific literature on health warning labels and consumer information on alcohol products. Alcohol health warning labels are defined in this review as labels found on alcohol containers and that include text and/or images (or symbols) about the health risks associated with the consumption of alcohol, or information pertaining to standard drinks or low-risk drinking guidelines. A combination of the following terms was used: alcohol, health warning, warning label, front of package message on harms. The search strategy is summarized in Table 1.

We include studies that referred to warning signs or posters in restaurants, bars or alcohol retail outlets, or warnings on alcohol advertisements, but only when these studies focused on health warnings located on alcohol containers. We did not include studies that focused only on nutrition labelling on alcohol bottles. Furthermore, studies were eligible for inclusion in the study if they:

- Focused on alcohol, or alcohol in combination with other drugs, involving warning labels or health messages on the bottle, can, or container;
- Presented messages related to standard drinks or low-risk drinking guidelines;
- Presented primary data from research; or
- Studied public opinions on alcohol warning labels.
- On the other hand, studies were excluded if:
- They were a commentary, editorial, letter or opinion piece about tobacco, cocaine, cannabis, food, or other drugs; about drinking behavior with no reference to warnings or health messages; about various alcohol-related harms with no reference to warnings or health messages;
- They focused exclusively on alcohol warning signs posted in licensed premises or other places that sold alcohol.

Literature reviews were saved in a separate folder and the data were used to support the findings from the primary research studies. 

**Figure 1:**
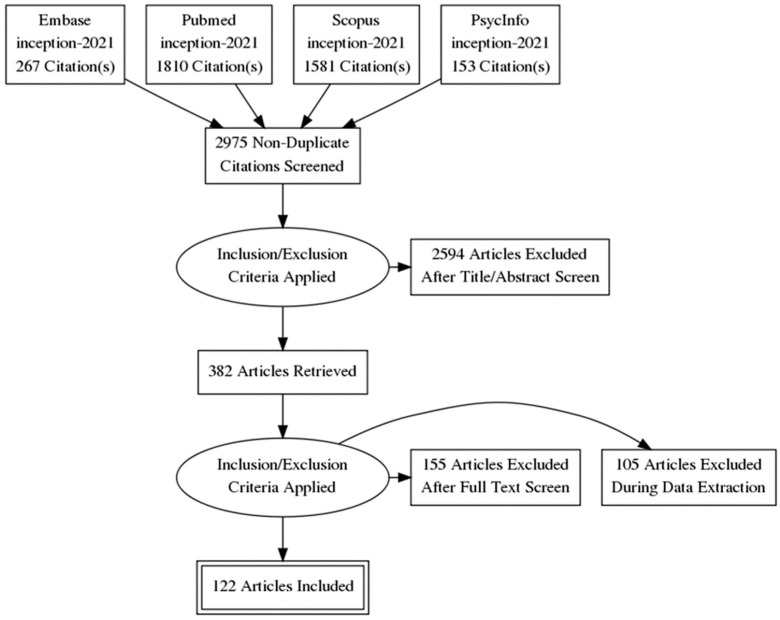
Preferred Reporting Items for Systematic Reviews and Meta-Analysis (PRISMA) flow diagram.

The search strategy was run by ER on Pubmed, PsycINFO, Embase, and Scopus in April 2021, and 3811 unduplicated abstracts were identified and filed. Abstracts published in English, Spanish and Portuguese were included. The results were imported, deduplicated and analyzed in Covidence, a web-based literature review management program. The abstracts were screened by two team members (ER and NG), and 382 were identified for further examination. Two team members assessed each abstract and 232 were selected for full-text assessment and data extraction by the two reviewers. There were an additional 150 papers where there was disagreement between the two reviewers and a third reviewer (IR) was the tiebreaker. Eventually, 227 were selected for data extraction. During data extraction, 105 papers were excluded. Finally, 122 papers were included in this rapid review. See Figure 1 for further details about the data screening process.

Finally, reference lists of remaining papers were hand-searched for additional relevant studies, but no additional articles were located. Figure 1 reports the final number of selected studies after completing the appraisal in a Preferred Reporting Items for Systematic Reviews and Meta-Analysis (PRISMA) flow diagram [41].

ER and NG extracted data from five papers and the findings were similar. The remaining papers were divided equally between ER and NG and the following data were extracted from the studies: first author and year; where and when the study was done; goal; design/type of study; source of data/number of respondents; focus; specific population; impact/outcome; and recommendations regarding AWLs. Narrative synthesis was used to analyze the results. 

A streamlined version of the extracted data is presented in the Supplementary Tables that are linked to the Results. All three authors contributed to preparing the Supplementary Tables and finalizing the manuscript. For the Supplementary Tables, the rows in each table are organized from the date of publication with the oldest first to the most recent.

## 3. Results

Many of the studies were conducted in the United States (n = 58) with others in Australia (24), Canada (23), the United Kingdom, England or Scotland (19), and Italy and France (5). There were 1–3 studies based in each of the following countries: Germany, Nigeria, New Zealand, Spain, Luxembourg, India, Israel, Thailand, Trinidad and Tobago, and China; two in Europe and nine with a global focus.

While some studies had multiple foci, we grouped them by a main theme in each publication. More details about each study are provided in the Supplementary Tables, which are labelled the same as the sub-sections of the Results.

The foci varied from: design of warning labels; message content of AWLs; perceptions of and awareness about AWLs; impact on adolescents; impact on young adults or college students; impact on adults and general populations; pregnancy and AWLs; AWLs and cancer; public support for alcohol warning labels; alcohol industry messages; and policy analysis and AWLs. 

Furthermore, the grouping is not organized chronologically by year of publication; for example, a number of the public opinion studies appeared 30 years ago, and those focusing on cancer more recently. This literature reflects a wide range of settings, contexts and designs, including surveys based on representative samples, convenience sample surveys, focus groups, case studies, in-person and on-line experiments, and quasi-experimental studies.

We also assessed the conflict of interest statements in the manuscripts. We randomly selected 32 publications, as well as those from the alcohol industry and policy analysis sections that were not included in this random sample. These 42 papers were examined to determine what the authors stated with regard to possible conflicts of interest. The results were: 18 made no mention of conflicts of interest; 19 stated there were no conflicts of interest; and five stated there were no conflicts of interest and then listed funding or affiliation with a research organization. Included in this group of 42 was one paper which declared ‘no conflict’ but noted partial funding from a vine and wine organization.

### 3.1. Warning Label Requirements 

#### 3.1.1. Research on AWL design

Especially in recent years, there has been research into the design of AWLs, which refers to a number of dimensions, such as where the label is located on the alcohol container, e.g., the front or back of the container, the space devoted to the label, and whether it includes text, images or both. While in some cases the research on label design also overlaps with the message content, we have summarized them separately. Although research on label design does not provide definitive conclusions, it points to several characteristics of AWLs that will increase the attention paid to them by those viewing them: a label on the front; a combination of text and image warnings; enhanced pictorial representation; and large warning labels—although the research does not specify size.

Nineteen papers were identified with label design as a primary focus. These papers involved a combination of experimental studies and eye-tracking designs that were not necessarily representative of the general population (Supplementary Table S1). Highlights from a number of these papers are noted below.

With regard to placement of the labels, according to an experiment by Godfrey et al., messages were more likely to be noticed if they were on the front of the container and not the neck [42]. Nevertheless, Annunziata et al. [43], in their on-line survey experiment of Italian and French generation Y consumers, reported they preferred no AWLs, then on the back where it was less visible, and neutrally framed messages. 

Several studies examined the size of the label. An on-line experiment by Al-hamdani and Smith [44] received partial support for the conclusion that extra-large warnings received lower product-based ratings than medium-sized, as did plain packaging of alcohol products. Coomber et al.’s [45] focus group study found that warning size and placement stood out from product labels but did not think that they would deter them from risky drinking. Larger warnings were assessed as having more potential impact, as noted in an on-line experiment in Nova Scotia [46] as well as an eye-tracking experimental study in Australia [47]. In a UK-based study, Wigg et al. [48] noted that pictorial health warnings were associated with significantly higher fear arousal as well as greater intentions to reduce alcohol consumption.

In an experimental study of Spanish adults, Sillero-Rejon et al. [49] found that there was greater attention paid to larger health warning labels, and that differences in visual attention depended on the alcohol by volume (ABV) being present. An experimental study in Thailand [50] found that warning pictorials could be used on alcohol containers in order to evoke affective states.

Some research considered both text and image content. In a randomized controlled trial by Al-hamdani and Smith [46] that assessed four labelling conditions, plain labelling and combined text and image warnings seemed to have a stronger effect on alcohol consumers than the use of texts alone. In-depth interviews with young French people by Dossou et al. [51] found that, for the majority, recall and awareness were higher for the text warning than for the pictogram on the advertisements, and most said the alcohol warnings were not effective in changing behavior. In an eye-tracking study by Monk et al. [52], regardless of content, positive outcome expectancies with regard to alcohol-related beliefs were associated with individuals attending to the imagery for longer time periods. 

A survey of Italian adults found that the majority of wine consumers were interested in health warnings on the wine label and tended to prefer a logo as well as a claim [53]. A randomized controlled trial in the United Kingdom by Vasiljevic et al. [54] reported that the total amount of drink consumed increased as the label on drink denoted successively lower alcohol strength. 

UK-based research by Zahra et al. [55] examined the cognitive processing of emotive pictorial warning. In the first study there was no difference in reasoning accuracy between positive and negative content. However, fewer correct responses followed exposure to negative general-health messages. The second study suggested that when a warning involves the potential consequences of drinking alcohol, accuracy is improved when the content is negative. The authors concluded that negative imagery should be used with caution, since it does not appear to be generally beneficial in influencing the behavior of those viewing the label. Clarke et al.’s [56] on-line experiment showed that image and text AWLs reduced the odds of selecting an alcoholic drink compared with text only, but increased the odds of selecting an alcoholic drink compared with image-only AWLs. Gorn et al. [57] conducted a study involving students in a Canadian university who designed seven labels that all had personal references (“you” or “your”), and presented consequences of drinking and driving that were realistic.

Finally, Gold et al. [7] reported on a large-scale parallel randomized controlled trial (n = 7516) in England which assessed low-risk drinking guidelines and health warnings. Labels with enhanced pictorial representations of alcohol content improved knowledge and understanding of the UK’s low-risk drinking guidelines compared with industry-standard labels; health warnings did not improve knowledge or understanding of low-risk drinking guidelines. Designs that improved knowledge mostly had the low-risk drinking guidelines in a separate statement located beneath the graphics.

#### 3.1.2. AWL Message Content

In 12 studies, the primary focus was on the content of the message on the AWL (Supplementary Table S2), and a number are highlighted here. Content refers to either the text of the message, images or pictorial content, or both. Outcomes assessed varied, including awareness of risks or harms from alcohol, intentions to change drinking behavior, or actual changes in behavior. A number of different messages had an impact on respondents’ attention to the AWLs. These included messages about risks for drinking among youth and birth defects and alcohol use. In two studies, health warnings with strong causal language were considered as being more effective in achieving an impact than those with equivocal language.

A range of specific message themes are assessed in this research. Two studies, based on 273 US university students [58,59], assessed five warning labels: pregnancy and birth defects; impairment to drive a car; risk of hypertension, liver disease, or cancer; the danger of alcohol in combination with other drugs; and warnings that alcohol is a drug and may be addictive. The first study by Andrews et al. [58], conducted prior to the implementation in 1989 of the US Alcohol Labelling Act, found that label believability was greater to the occasional/non-users than among frequent users. In the second study [59], pair-wise comparisons indicate that the birth-defects label was perceived to be significantly more favorable than the other four labels. The authors found net support arguments to mediate the relationship from attitudes toward drinking to attitudes toward the warning label.

Annunziata et al. [60] also used an on-line survey platform to assess five different warning messages among respondents from Italy, France, Spain and the US. Consumers from all countries tended to assign high utility scores to “ban alcoholic beverages to children under 18/21 years of age”, and “do not drive after drinking”.

Pechey et al. [61] used an online survey of 5528 participants to assess the effectiveness and acceptability of image and text warning labels. Image and text health warning labels depicting bowel cancer showed the greatest potential for reducing selection and consumption of alcoholic drinks and energy-dense snacks, although they were the least acceptable.

However, Kersbergergen and Field [62] conducted a study of UK students using an eye-tracking technique. Alcohol consumers allocated minimal attention to warning labels on alcohol packaging, and even if their attention was directed to these warning labels, this had no impact on their drinking intentions. The lack of attention to warning labels, even among people who actively wanted to cut down, suggests that there is room for improvement in the content of health warnings on alcohol packaging.

Some research considered multiple components of the message. A laboratory eye-tracking study based in Cali, Colombia [63] involved 114 adult participants with approximately 100 wines. There were significant differences between men and women, with men showing a greater effect on their purchase intention by considering the three components of the label: the product’s origin, the nutritional information, and the health warning on the label in relation to consumption. Clarke and Rose [64] conducted a mixed-methods experiment with 162 young adults in Liverpool, with some shown a labeled glass with unit markings and a health warning, and others a non-labeled glass. Multi-level modelling showed that there was no significant main effect, indicating that participants did not differ in their drinking by glass type. Most noticed the unit and warning labels and did not believe that these influenced their intake. There was no significant main effect on the condition on change in alcohol urge. Most participants highlighted that the information could be of use for monitoring consumption, but that it was unlikely to be used to consume within the guidelines. Another key point raised in the focus groups was that participants perceived unit information as confusing, and guidelines as unrealistic. It is possible that presenting different types of information on labels may have an increased impact on behavior.

Multiple messages were also assessed by Hobin et al. [65] in an online survey of 2016 respondents. They examined the efficacy of alcohol labels with standard drink (SD) information and Canada’s Low-Risk Drinking Guidelines (LRDG). Between-groups experimental design was used. Respondents were asked to estimate three primary outcomes: the amount in a standard drink, the number of standard drinks in an alcohol container, and the number of standard drinks to reach Canada’s LRDG. Participants exposed to larger labels with both SD information and the LRDG were more likely to correctly respond to all three primary outcomes compared to those exposed to smaller labels. Furthermore, the results indicated that labels with SD and LRDG information facilitated more accurate estimates of alcohol consumption and awareness of safer limits across different beverage types.

There are varying results on how “strong” the warning language should be. Sillero-Rejon et al. [49] conducted a laboratory experiment (n = 128) with a control group in Spain. They concluded that in comparison to moderately-severe health warnings, highly-severe health warnings increased avoidance and reactance, and were perceived as more effective and increased motivation to drink less. Hall et al. [66] conducted a randomized experiment on a convenience sample of 1413 US adults, with 3/4 of the respondents indicating that health warnings with strong causal language were the most effective. In contrast, Annunziata et al. [67] examined the influence of alcohol warning labels on wine and beer consumption, based on 394 persons from Italy and 265 from France. For those choosing beer, the respondents preferred to be informed about the possible negative consequences, but with a neutrally framed message. Those choosing wine preferred no warning message.

### 3.2. Perception and Awareness of AWLs 

There were 13 studies that focused primarily on perception and awareness of alcohol warning labels. For further information, refer to Supplementary Table S3. There are some generalizations that emerge from the studies discussed below. Awareness of the existence of AWLs tended to be greater among certain groups, such as drinking drivers, impaired drivers, and those of younger age. In other words, the messages appeared to be stimulating the attention of some sectors of high-risk drinkers. Some research found that viewing alcohol warning advertisements reduced negative implicit attitudes among heavy drinkers. However, in the Yukon quasi-experiment, AWLs increased awareness of low-risk drinking guidelines and drinking limits compared with the comparison site.

Scammon et al. [68] evaluated the 1989 US legislation on alcohol warning labels, focusing on surveys of Utah households, three waves before and three waves after the legislation. There was no evidence that perceptions of the risks of alcohol abuse or behavior regarding alcohol consumption had been altered in response to the warning labels.

However, there is evidence that heavy drinkers pay attention to AWLs. In the US, Parker et al. [69] noted that both drinking drivers and impaired drivers (based on their reports) were more likely to recall the US labels or their content in the post-introduction period. Brown et al. [70] recruited 373 UK participants aged 18–40 through an online research agency. They were randomly assigned to one of three advertisements: promoting alcohol, warnings about alcohol, or unrelated to alcohol. Viewing alcohol-promoting advertisements was found to have a cognitive impact on heavier drinkers, increasing positive and reducing negative implicit attitudes towards alcohol. Viewing alcohol-warning advertisements reduced negative implicit attitudes towards alcohol in heavier drinkers, suggestive of a reactance effect.

There is also indication of an impact on youth and young adults. Research by Parsons et al. [71], with a random sample of 482 homeless persons from Cook County, found that younger age and more frequent drinking were associated with greater label awareness and familiarity with the content. de Turck et al. [72] examined the effectiveness of alcohol beverage warning labels based on 198 university students receiving 5 different warning messages on a computer monitor and then being asked to recall information. The product was perceived as most dangerous when they attempted to memorize the warning and the signal word was in red, and females rated alcoholic beverages more dangerously than males. However, participants who consumed alcoholic beverages more frequently perceived these beverages as much less dangerous than did other respondents.

Several US-based studies focused on Hispanic populations. Marin [73] reports on research involving Hispanics in San Francisco. In a cross-sectional survey of 1204 Hispanics, drinkers of alcoholic beverages reported the highest levels of warning signs and messages, and their length of exposure produced greater levels of AWL awareness; however, less acculturated, Spanish-speaking Hispanics were less likely to report being aware of the warnings. A study by Marin and Gamba [74] involved a total of 4661 randomly selected samples of Hispanics; they found moderate cumulative effects involving increased awareness of the product label with a warning with continued exposure to product warning messages, and in particular the “Alcohol and Drinking” message. During a one-year period between 1991 and 1992, 777 Hispanics and 234 non-Hispanic Whites were surveyed; both samples showed an increase in awareness of warning labels for both beer and wine [75].

Two experiments by MacKinnon and Lapin [76] did not support the “boomerang effect”: that is, exposure to the warning led to higher positive perceptions of the product listed. 

In some cases, there were gender differences. Coomber et al. [77] conducted an online research panel of 1061 Australian respondents. Those who were aware of warning labels were more likely indicate “definitely true” to questions about harm to unborn babies and cirrhosis of the liver. The authors also noted that almost all participants had heard of a standard drink and 80% recognized the logo. Overall, 73% of participants supported the inclusion of information about recommended daily guidelines to reduce high-risk health effects on labels for alcohol products. Women were significantly more likely than men to support the inclusion of such information, as were those with a tertiary or higher level of education and low-risk drinkers.

In a few cases, AWL information was combined with information on low-risk drinking guidelines and standard drink volume. The quasi-experiment conducted in Yukon, Northern Canada examined awareness of low-risk drinking guidelines [78]. With the introduction of alcohol warning labels in the intervention site, awareness of the guidelines increased by 2.9 times and knowledge of drinking limits also increased in contrast to the comparison site. Furthermore, participants in the intervention site, in contrast to the comparison site, had greater odds of reporting using standard drink information to purchase alcohol.

### 3.3. Impact on Different Populations

#### 3.3.1. Adolescents and AWLs

Six studies were identified where the focus was on adolescents (Supplementary Table S4). In three cases, the assessment was of the US-mandated alcohol warning label of 1989. The studies based on the US AWLs noted that adolescents were aware of their existence. Those who drank less alcohol were more likely to believe the risks on the AWL. Awareness of a product label with a warning was greater among current drinkers, and in one large scale study, pictorial messages had more potency to elicit emotions. 

MacKinnon et al. [79] conducted a before-and-after survey of 12th grade students. They noted that after the US labels appeared, more adolescents reported seeing them, were aware of them, and recognized the risks that were noted on the label. Between the 1989–1990 and 1994–1995 school years there was an increase in awareness of the AWLs, exposure and memory among high school students [40]. Nohre et al. [80] conducted a survey of 1211 to 3174 grade 12 students between 1989 and 1992. They reported that students who were female, had higher grades, attended religious services more frequently, watched less television, and drank less alcohol were more likely to believe the risks on the warning label.

In a UK survey of 3399 adolescents, Critchlow et al. [81] reported that awareness of seeing alcohol health warnings or other product information was greater among current drinkers versus non-drinkers, high-risk drinkers versus lower-risk drinkers, and susceptible never-drinkers than non-susceptible never-drinkers. Research by Moss et al. [82] recruited 94 underage drinkers from colleges in the UK in order to examine the effects of exposure to health messages on attitudes towards drinking and drunkenness, and intentions to drink and get drunk. In contrast to possible expectations, active exposure to responsible drinking messages (RDMs) led to more positive attitudes toward drunkenness, while passive exposure led to more negative attitudes. The authors caution against using RDM interventions amongst underage drinkers.

Morgenstern et al. [83] conducted a survey-based experiment, and noted that among 9260 German adolescent students, pictorial messages had more potency to elicit emotions, at least among recipients that already had experience with alcohol use.

#### 3.3.2. Young Adults and College Age Respondents and AWLs

Overall, 14 reports focused on this population (Supplementary Table S5). There was some evidence of a boomerang effect, with some respondents giving higher ratings on the benefits of alcohol products when they saw the AWLs. The findings on standard drink information are not consistent—one study indicated that it was used by respondents to increase their consumption and in another study it was linked with reduced consumption. In one study, AWLs that were large, with combined (text and image) warnings with specific messages on the front of the packaging, were considered the most engaging and potentially effective.

Several studies focused on the content or tone of the messages. Andrews et al. [84] reported that messages about birth defects and driving impairment were perceived to be most believable. Garretson and Burton [85] noted that there was high awareness of these risks. DeCarlo et al. reported that two messages pertaining to not drinking during pregnancy or alcohol-related road deaths were ranked as most intense and most effective with regard to this adolescent population [86].

A German sample of 40 college-aged students [87] indicated that warning labels that contradicted positive alcohol outcome expectances had greater effectiveness than warning labels that illustrated health-damaging consequences of alcohol consumption. Jarvis and Pettigrew [88] conducted an Australian-based study using a web-based sample in order to assess the relative influence of alcohol warning statements on young drinkers’ stated choices. In general, negatively framed messages had the greatest influence on the higher-consuming classes. 

In a small-scale experiment of university students in Germany and Luxembourg, Krischler and Glock [89] noted that warning labels presented as statements had no influence on individual and general outcome expectancies and drinking intentions. Warnings posed in the form of questions showed some effectiveness, as they increased individual negative outcome expectancy perception.

The US 1989 alcohol warning label was the focus of the research of Malouff et al. [90] based on four studies. They concluded from the students’ reports that a more conspicuous design would make it more likely that drinkers would notice, read and recall the warning.

In some cases, there was variation in outcomes depending on drinking levels. According to MacKinnon and Fernaughty, heavier users, the group at higher risk for problems described on the warning label, were also most likely to retain the content of the AWL [91]. Additionally, those who drank more alcohol may have selectively reduced their exposure to AWLs, and those who viewed themselves as health-conscious had a greater willingness to read AWLs [86].

An experimental study by Snyder and Blood [92] included participants viewing a set of slides with or without a warning and advertisement. They detected a boomerang effect. Contrary to the legislative intent of the warnings, the young drinkers in the experiment actually displayed higher ratings of the benefits of alcohol products when they saw the warnings.

An experimental assessment of 274 university students in Australia and US by Creyer et al. [93] examined how two different alcoholic beverage health warnings on a fictious brand of beer influenced alcohol-related risk perceptions, attitudes and intentions, and characteristics of problem-drinking behaviors of binge and non-binge drinkers. The standard warning label used in the US and the “Alcohol is a Drug” warning were assessed. For binge drinkers, the US label had lower risk perceptions than the “Alcohol is a Drug” warning, thus reinforcing stronger warnings.

The information on a standard drink produced divergent results in two studies. In a focus-group study, young adults used standard drink information to maximize their alcohol consumption [94]. However, in a UK study by Armitage and Ardenm involving two experiments, standard drink information was capable of significantly reducing alcohol consumption [95].

Some studies examined students’ awareness and perception of risks related to drinking. For example, Annunziata et al. [96] analyzed 385 interviews with University of Naples students aged 18–30. They found that the respondents were reasonably aware of the risks linked to excessive alcohol consumption and considered the spread of alcohol consumption among their age group a serious matter. They also wanted further campaigns via TV, social media or warning posters in licensed premises. However, the students were confused about the concept of moderate alcohol consumption and had scant knowledge of the thresholds imposed by the law to limit driving after drinking.

Finally, in a study conducted in Glasgow, Scotland by Jones et al. [97], focus groups were used to explore 50 young adult drinkers’ awareness and perceptions of health information and messaging displayed on alcohol packaging. Those who supported introducing warnings maintained that they should be noticeable, fact-based and relevant to real life. Most participants thought that including a range of rotating warnings on packaging (e.g., short- and long-term risks) could help to reach more consumer profiles and prolong impact. Most thought that these warnings could increase awareness of alcohol-related harms, particularly for younger or potential drinkers. Large, combined (text and image) warnings with specific messages on the front of packaging were considered most engaging and potentially effective. Most groups thought that the cancer warning would be more believable, informative and effective if the types of cancers caused by alcohol consumption were specified, which could help to improve awareness and knowledge of the alcohol–cancers link.

#### 3.3.3. General Populations and AWLs

We identified 14 of the studies involving evaluations of general populations’ awareness or knowledge of the existence of AWLs, or actions taken after seeing them (Supplementary Table S6). A number focused on assessments of the impacts of the 1989 AWLs introduced in the US. AWLs were noticed by those at risk of the alcohol-related hazards noted in the labels, and who had been at risk of drinking and driving, thus suggesting that members of an intended target audience were paying attention to them. Conversations about drinking during pregnancy were more likely among those who had seen the AWLs. Supporters of the AWLs indicated that they had affected their alcohol consumption. More recent research noted that, after respondents were exposed to a statement about a health risk factor associated with alcohol, there was an increase in belief in the statement. The most compelling research to date comes from the Yukon experiment, which found that respondents increased their awareness of cancer as a risk factor when recalling the AWLs, reported drinking less after being exposed to the AWLs, and that alcohol sales declined during the intervention. 

The research by Greenfield, Kaskutas and Graves assessed the links between the implementation of the US alcohol warning label and adult respondents and their behavior. Their results are based on a series of surveys representative of the general population, including surveys in the Canadian province of Ontario which did not have any AWLs. Among the US respondents, warning labels were noticed by many of those at risk of hazards discussed in the labels [98]. There was a higher penetration of AWL awareness among heavy drinkers, young men and young women. Respondents who saw the label were more likely to talk about alcohol and pregnancy and about drinking and driving [99]. Exposure to the warning was predicted primarily by consumption pattern, but also was associated with a personal salience factor, that is, ever having drunk alcohol so as to be at risk of harm when driving [100].

Kaskutas and Greenfield [101] concluded that respondents who probably saw the warning labels were significantly more likely to report several behaviors indicative of heightened awareness of, and caution regarding, the hazards of drinking and driving and of drinking during pregnancy, hazards that are both included on the warning labels. Kaskutas [102] examined differential perceptions of alcohol policy effectiveness. Heavier drinkers were significantly less likely than moderate drinkers to believe that the AWLs had affected their own drinking. Many of the label supporters said that it had indeed affected their drinking (59%), as compared to its non-supporters, among whom only 18% said it had affected their drinking. Those who believed the warning label had affected their own drinking (other variables controlled for) were six times more likely to support the label policy than those who did not believe it had affected their drinking, and this belief was the strongest predictor of support for that policy, even more so than consumption.

Greenfield et al. [103] noted that the proportion of reported conversations about drinking during pregnancy was higher among those who had seen the labels. There were also modest effects of precautionary behavior: reports of limiting drinking for health reasons showed a positive association with label exposure, increasing with time. Research by Tam and Greenfield [104], involving surveys before and after the US label was introduced, found that message recall and intervening—that is, to deter another person’s drinking and driving—were related.

An Australian on-line survey [105] concluded that, for all conditions except liver damage, the extent to which alcohol was believed to be a risk factor for a specific chronic disease was significantly greater after respondents were exposed to a statement presenting information advising of such risk. The effect sizes associated with these pre- to post-exposure changes were large, especially for the statement “Alcohol increases your risk of diabetes”, followed by “Alcohol increases your risk of mental illness” and “Alcohol increases your risk of heart disease”.

There have been several publications on the quasi-experimental study conducted in the Canadian northern territory of Yukon. Thinking about labels increased to a greater extent than in the comparison site, as did talking with others about alcohol issues [106]. The percentage of participants that reported drinking less alcohol due to labels increased to a greater extent overall between waves one and three in the intervention versus the comparison site.

The authors noted that their study indicated that how health risks are communicated to drinkers is consequential. Strong health messages on alcohol containers changed how drinkers processed and responded to the information. The research team found that, in the course of the intervention period, there were statistically significant increases in all five key indicators among those exposed versus those not exposed to the alcohol label intervention [106]. 

Recall of the cancer warning label increased to a greater extent in the intervention versus the comparison site in the Yukon study. In addition, reports of drinking less because of the label increased to a greater extent in the intervention versus the comparison site [8,106].

In the Yukon study, there was an increase in knowledge of cancer as a risk in the intervention site and recall of the cancer warning. Those who recalled the cancer message had 2.3 times greater odds of knowing that alcohol can cause cancer [107]. Zhao et al. [8] tracked total per capital retail alcohol sales in Whitehorse, the capital of Yukon territory. Sales decreased by 6.31% during the intervention, and decreased among labelled products by 6.59%, whereas sales of unlabeled products increased by 6.91.%

### 3.4. Impact of Different Messages

#### 3.4.1. Pregnancy and AWLs

A number of studies [35] have considered pregnancy and AWLs (Supplementary Table S7). While the general focus was on pregnancy and AWLs, the specific focus varied from study to study, including awareness of alcohol as a potential risk factor during pregnancy, drinking behavior during pregnancy, or intention or actual change in behavior associated with information on the AWL. In many cases, the research is based on evaluations of the impact of the US alcohol warning label legislation of 1989. Awareness of the existence of AWLs was highest among young adult females. Pregnant women were more likely to recall the message on alcohol and pregnancy compared with non-pregnant women, and were more likely to correctly identify the effects of prenatal alcohol exposure. This research also identified some myths about alcohol and pregnancy; namely, that alcohol consumption is helpful, and that consumption of beer and wine during pregnancy is less dangerous than consumption of spirits.

Hankin et al. published seven papers based on large samples of African-American gravidas from an inner-city Detroit hospital [108,109,110,111,112,113,114]. Younger women were more aware of the label. Awareness of the warning label reached 78% by June 1993, which was about four years after the US label was introduced, and risky drinkers with more exposure to the label had higher level of awareness of AWL existence. Knowledge of the warning label increased three months after the implementation of the law; however, the decline in drinking was small. Between May 1989 and September 1991, the distribution of drinking during pregnancy remained stable.

Several other studies also focused on awareness of and knowledge about alcohol-related risks. Based on pre- and post-legislation US national surveys, Mazis et al. [115] noted that about 11% were able to identify the specific pregnancy hazard warning. Awareness level of these risks was highest among younger adults and “heavy” alcohol consumers.

Chambers et al. [116] surveyed 100 pregnant Latinas from San Diego County. More women who reported any drinking during the periconceptional period had heard of fetal alcohol syndrome and could correctly identify outcomes associated with prenatal alcohol exposure compared with non-drinkers.

Recall of AWL content was another theme. A study by Blume and Resor [117] involved 99 Mexican-American women who had been or were of child-bearing age. English language skills significantly predicted participants’ ability to remember health warnings on beverage containers. Furthermore, beliefs that drinking during pregnancy is helpful and not associated with liver and cognitive problems were significantly associated with higher alcohol consumption. In addition, the belief that drinking helps when pregnant, along with a reported history of drinking during a previous pregnancy, significantly predicted self-reported drinking during a most recent pregnancy. 

A random-digit-dialing survey of 1515 women of child-bearing age in Illinois [118] found that 28% of all women were able to recall information on pregnancy risk on alcohol beverage container warning labels. Three times as many pregnant women than non-pregnant women were able to recall the information. Pregnant women and younger women were significantly more likely than non-pregnant women and older women, respectively, to be able to recall information about pregnancy risks contained in alcohol warning signs.

Other research assessed the perceived value of the AWLs by pregnant women. Mayer et al. [119], using three waves of data collection from 400 Utah households, noted that the admonition against drinking during pregnancy was the most memorable portion of the warning. However, there was no evidence that the labels affected knowledge of the health and safety risks associated with alcohol or self-reported alcohol consumption. 

Parackal et al. [120] reported on a national sample of 1129 16–40-year-old New Zealand women. They assessed their views on warning labels on alcohol containers as a source of information on alcohol and pregnancy. Just over half of the women gave a high rating for the warning label as a source of information about alcohol consumption and pregnancy. Women below 30 years of age and who were of non-European ethnicity were more likely to give a high rating compared with older women and European women, respectively. A study in Italy by Bazzo et al. [121], which involved 694 parents or care-givers of children aged 0–2, assessed their reactions to an image and message about avoiding alcohol while pregnant. Overall, 84% of the respondents said that they remembered seeing the image. The image seemed to have a high impact in the majority of the respondents.

A study in France highlighted some myths about beverage type and pregnancy. Alcohol warning labels were introduced in France in 1991 and 2007. Dumas et al. [122] reported that five years after the labels were introduced, a large proportion of women believed that the consumption of beer and wine was less dangerous than spirits during pregnancy, even though the labels appear on every type of alcohol container.

Finally, Odeigah et al. [123] highlighted national differences in the prevalence of pregnancy warnings. They compared labels on 13 products in Nigeria and the United Kingdom. All product labels of alcoholic beverage brands produced in the UK contained at least two health warnings, while 36.4% of the labels had all four health warnings. Compared to Nigeria, products in the UK were more likely to have a “don’t drink when pregnant” logo (18.2% versus. 100%).

#### 3.4.2. Cancer and AWLs

Seven research papers have focused on cancer as a potential risk factor of alcohol consumption. In addition, the Yukon study [107], highlighted in Section 3.3.3 (above) used cancer as a key message in their warning labels, until this was blocked by the alcohol industry [4,124]. Further information on AWLs and cancer is provided in Supplementary Table S8. 

In less than 10 years, a number of studies have examined the impact of cancer messages as part of AWLs. Several studies highlighted that awareness of alcohol as a carcinogen was typically low. However, there was strong support for enhanced AWLs that included a reference to cancer as a possible harm from alcohol consumption. Respondents found favorable changes in alcohol consumption intentions even among high-risk drinkers. In one large-scale study, women and high-risk drinkers reported being more likely to reduce their alcohol consumption in response to all labels involving seven types of cancer. A combination of image and text warnings was found to reduce the odds of selecting an alcoholic drink. Knowing that alcohol can cause cancer increased the likelihood of supporting an AWL, and also increased support for alcohol pricing policies.

An international study by Winstock et al. [125] included data from 79,969 adult respondents from 29 countries/regions who reported the use of alcohol in the last 12 months. The fact that drinking less can reduce the risk of seven types of cancer was not well known, and yet was shown to encourage almost 40% of drinkers to consider drinking less. Women and high-risk drinkers were more likely to indicate they would reduce their drinking in response to all labels. Personal relevance was identified as a key predictor of individual responses. 

In the Yukon study, there was low knowledge (24.5%) of alcohol-related cancer risk at base-line among 836 liquor store patrons [9]. The results of five focus groups, reported by Vallance et al. [5], indicated that the majority of participants showed strong support for enhanced alcohol labels, with an emphasis on the consumer’s right to know about the health risks related to alcohol. Participants also preferred larger labels that included standard drink (SD) information, national low-risk drinking guidelines presented as a chart with pictograms, cancer health messaging and a pregnancy warning.

Several studies considered the acceptability and potential impact of having a cancer message in AWLs. A mixed-methods study by Pettigrew et al. [126] investigated the acceptability among drinkers of cancer warning statements for alcoholic beverages. An on-line survey of 2168 respondents and six focus groups was conducted with Australian drinkers. Overall, responses to the cancer statements were neutral to favorable, indicating that they are unlikely to encounter high levels of negative reaction from the community if introduced on alcoholic beverages. Positively framed messages, those referring to specific forms of cancer, and those using “increases risk of cancer” wording performed better than negatively framed messages, those referring to cancer in general, and those using the term “can cause cancer”. 

A national survey of 1680 Australian adults, also by Pettigrew et al. [127], investigated the effectiveness of six alcohol warning statements designed to increase awareness of the alcohol–cancer link. Pre- and post-exposure data were captured relating to respondents’ alcohol consumption intentions. Of the six statements tested, “Alcohol increases your risk of bowel cancer” produced the highest scores across all outcome measures. All statements produced favorable changes in alcohol consumption intentions, including among high-risk drinkers. 

Three further reports from Australia focused on the potential impact of messages about alcohol as a risk factor for cancer. Miller et al. [128] surveyed 1600 participants, and over 70% agreed that labels could raise awareness of and prompt conversations about the cancer risk associated with alcohol. May et al. [129] reported on seven focus groups, with participants challenging the legitimacy of the alcohol-related cancer messages. Ma [130] examined the role of pictorial warning labels (PWLs) which featured narrative content in communicating alcohol-related cancer risks. It was found that exposure to narrative PWLs significantly increased participants’ worry about, feelings of risk of, and perceived severity of harm of getting alcohol-related cancer. The author suggests that narrative PWLs are a promising strategy in informing consumers about the cancer risks of alcohol consumption.

Additionally, an on-line UK-based study by Clarke et al. [131] focused on health warning labels that communicated the risk of cancer. Image and text health warning labels reduced the odds of selecting an alcoholic drink compared with text-only health warning labels, but increased the odds of selecting an alcoholic drink compared with image-only. Furthermore, Blackwell et al. [132] conducted an online survey of 1844 respondents in Bristol, noting that participants reported higher motivation to drink less after viewing both cancer messages and negatively framed messages.

The Yukon project involved enhanced labels, including one version with the large text “Alcohol can cause cancer, including breast and colon cancer”. A baseline survey of 836 selected liquor store patrons at the intervention site [9] found that knowledge of alcohol-related cancer risk was 24.5%, limited ability to calculate a standard drink was 29.5%, and that under half had knowledge of daily or weekly low-risk drinking guideline limits. 

Following the quasi-experiment, recall of the cancer warning label was increased to a greater extent in the intervention versus the comparison site, and reports of drinking less because of the labels increased to a greater extent in the intervention versus the comparison site [107]. In addition, those who recalled the cancer message had 2.3 greater odds of knowing alcohol can cause cancer, and those who know alcohol can cause cancer were 1.6 times more likely to support health warning labels relative to those who did not know this [133]. Furthermore, two waves of longitudinal surveys in Canada tested the impact of warning labels on consumers’ awareness of alcohol-related harms such as cancer [134] before and after labels were introduced in Yukon, the intervention site. Increasing individual-level knowledge of alcohol as a carcinogen was associated with higher levels of support for alcohol pricing policies in this study, specifically setting a minimum unit price per standard drink of alcohol. 

### 3.5. Public Opinion on Alcohol Warning Labels

In general, respondents typically indicated support for AWLs, but the results were equivocal about whether the labels would be expected to change the behavior of those who saw them. These conclusions are based on several large-scale surveys of representative samples of adults, mainly limited to surveys conducted in Canada and the United States (Supplementary Table S9).

A US-based survey by Hilton and Kaskutas [135], conducted before the US AWL was implemented, found that 87% of respondents supported AWLs, with 92% among women, and lower support among those aged 30–39. Even the majority of heavier drinkers supported warning labels. However, 89% projected that warning labels would not have much effect “as far as really heavy drinkers are concerned”. A follow-up study [136], conducted 18 months after the US AWL was implemented in 1989, noted that 91% favored the AWL policy compared with 87% in 1989. Abstention, having been exposed to the warning label, and a person’s sex (women) were associated with support. Additionally, more than 50% of respondents thought that an AWL was an effective way to change people’s behavior, in contrast to the previous survey [135].

Focusing on Ontario, Canada, Room et al. [137] noted that between 1989 and 1991 there was substantial net change in favor of AWLs even though Canada did not have AWLs. Furthermore, a 1998 survey by Anglin et al. found that 73% of Ontario adults said that AWLs were a good idea, with women and those with lower education being more favorable [138]. Ontario based surveys in 2000 and 2002 [139] reported that 68% of respondents said yes to having warning labels on alcohol beverage containers, with less support among frequent drinkers and those with higher Alcohol Use Disorders Identification Test (AUDIT) scores. Giesbrecht and Greenfield [140] compared the US and Canada, using representative samples of the population, reporting that in Canada 76% supported AWLs, the third highest of 11 alcohol policy items considered, and in the US 88% supported AWLs, the highest item, tied with education as an intervention.

Public opinion on AWLs is also reported in studies from several other countries. In a large-scale survey, Weiss et al. [141] found that Arab participants in Israel were more in favor of AWLs on alcohol containers than were Jewish respondents. An Australian study by Thomson et al. [142], based on mixed methods involving a cross-sectional survey and six focus groups, noted that 80–90% of those surveyed supported a range of information that could be mandated by government authorities for inclusion on labels, including health warnings.

Maharaj et al. [143] used a cross-sectional convenience sample. They found high support among respondents from Trinidad and Tobago for several components of health warning labels, including: placing more prominent warning labels on products displaying alcohol content (87.2%); placing more prominent warning labels on products showing harmful effects (88.5%); and increasing taxes on alcohol sales (87.7%).

Finally, Dekker et al. [144] conducted a web-based survey of 1000 adults per country, focusing on Australia, Canada, China, India, New Zealand, the United Kingdom and the United States. They considered 14 policy initiatives. Support was highest for labelling requirements, especially pregnancy warnings, but being a drinker was negatively associated with support for policies associated with AWLs.

### 3.6. Alcohol Industry and AWLs

We identified 10 studies with health messages associated with the alcohol industry or alcohol industry activities (Supplementary Table S10). The AWLs sponsored or prompted by the alcohol industry tended to have limited efficacy, were small in size, were voluntary and had low recall. In some cases, the intent of the messages was confusing to respondents.

There was research into recall of industry-sponsored messages and the impact on awareness of the harms and risks of alcohol consumption and intention to change behavior. Coomber et al. conducted a survey of 561 Australian adults who were assessed with their recall and recognition of “get the facts” and alcohol warning labels, and use of the DrinkWise website [145]. Participants who engaged in more frequent binge drinking, those who consumed alcohol directly from a can or bottle, and participants who supported the use of health warning labels were all significantly more likely to be aware of alcohol warning labels. Older participants were significantly less likely than 18–24-year-old participants to be aware of any of the warning labels. Older drinkers and females were less likely to be aware of the warning labels and visit the website, respectively. This general low level of warning label recall could be attributable to one-third of Australian alcohol products displaying a warning label, usually taking up less than 5 % of the label and on the back of the product.

In an eye-tracking study using US high school students, the beer advertisement, containing a voluntary message encouraging responsible drinking, had limited warning [146]. In focus groups of Australian university students, the voluntary alcohol products associated with DrinkWise were assessed [147]. They perceived them to be unconvincing and did not deter them from drinking to excess. 

There are indications that the messages promoted by the alcohol industry are unclear or not convincing. Both the social aspects and public relations organizations (SAPRO) associated with the alcohol industry developed ads (DrinkWise in Australia) and industry-developed adult-targeted campaigns (Budweiser in the US). The research by Jones et al. examined how these responsible drinking campaigns were perceived and interpreted by the general public [148]. The terminology used was found to be ambiguous, and messages were interpreted to have a wide range of different meanings, including some apparently unrelated to alcohol. In southern Italy, focus groups assessed two responsibility ads produced by the Heineken Company [149]. The study showed weak efficacy and interviewees failed to remember the existence of “drink responsibly” messages.

Research has also indicated variation in what type of messages the alcohol industry is willing to support. Tinawi et al. [150] examined a sample of 59 low-cost local and imported beers, wines and ready-to-drink alcoholic beverages available in New Zealand in 2016–2017, where warning labels are voluntary. They noted that 80% had pregnancy-related warnings, 73% had industry-led warnings, e.g., “responsible” consumption, and 19% had warnings about drink driving or heavy machinery. The warnings were small and covered between 0.21% to 0.12% of available space. 

The UK Public Health Responsibility Deal on alcohol labelling—a voluntary arrangement between the alcohol industry and government—was assessed as not being fully met [151]. Based on an analysis of the UK’s 100 top-selling brands, the labelling information that was included was considered to frequently fall short of best practice, with fonts and logos smaller than would be accepted on other products with health effects. 

There are also industry initiatives to thwart or block initiatives to put AWLs on alcohol containers. In Australia, various sectors of the alcohol industry used several tactics to delay the introduction of mandatory alcohol health warning labels, including: questioning the rationale and evidence base for labels; arguing that they will cause damage to public health and the economy; lobbying and seeking to influence the government and political representatives, including through monetary donations; and introducing their own voluntary labelling scheme [152]. Vallance et al. [153] examined media articles in Yukon and Ireland and found that similar arguments were used by the alcohol industry, including distorting or denying the evidence that alcohol causes cancer.

However, Gleeson and O’Brien [154] assessed the Trans-Pacific Agreement (TPP), and they found that the rules do not impede the right of governments to mandate AWLs.

### 3.7. Alcohol Policy Analysis and AWLs

Three papers were identified that examined AWLs and policy issues (see Supplementary Table S11 for more information). Lemmens et al. [155] did not find a dramatic increase in alcohol-related issues in the print media from 1985 to 1991, covering the time when US AWLs were introduced in 1989. An analysis of alcohol marketing and AWLs in Canada by Wettlaufer et al. [156] found that, as of 2017, no province had implemented AWLs. However, two northern territories have “stick on” AWLs. A study involving a general population sample, as well as policy influencers, in the Canadian provinces Alberta and Quebec [157] found strong support for information-based policies, with the exception of warning labels.

## 4. Discussion 

While there is extensive research on the topic of alcohol health warning labels, this review demonstrates that existing research is narrow in terms of scope and focus, and that most studies have focused on a few countries. Of the 122 original papers that we examined, the majority were based in the US, Australia, Canada, the United Kingdom, and Italy. 

There are 24 countries in the Americas, not including the United States, that have mandated or voluntary labels or alcohol health warnings. For the most part, these alcohol-warning codes in numerous countries of Central America, the Caribbean and South America have not been assessed with regard to their acceptance by the population and impact on knowledge, attitudes, intentions or drinking behaviors. Some years ago, Stockwell [35] identified six countries that had alcohol warning labels: India (State of Assam), Portugal, South Korea, Taiwan, Thailand, and Zimbabwe, that are not included in those noted by Giesbrecht and Rios [11].

Furthermore, Neufeld et al. [12] report on existing warning labeling legislation in 10 countries in the Commonwealth of Independent States (CIS). These authors did not find a single study “that documents the link between implementation of health warnings and attitudes toward drinking, awareness of the risks, and/or behavioral change.” In other words, there are excellent unrealized opportunities for documentation and evaluation.

Our review identified a range of designs and methods, including: cross-sectional surveys, repeated surveys of representative samples, surveys based on available samples, focus groups, and various on-line and in-person experiments. 

There were few randomized controlled trials, and an occasional quasi-experimental study, with the numerous publications on the Yukon project (e.g., Hobin et al. [133]) being exemplary. Furthermore, this project reported an impact on alcohol sales [8]. 

As illustrated in the Results there are a range of themes under the umbrella of alcohol warning labels, including, to mention several: public opinion about whether or not there should be AWLs; awareness of or perception of risk associated with AWLs; how viewers respond to them (e.g., conversation, intentions to change behavior, change in drinking behavior); what features of design and message content are most potent; how sponsors influence the perception of their validity; and whether the AWLs are voluntary or mandated. Not all methods or designs are ideal for each topic. Several suggestions for future work follow.

For research questions that consider public opinion on AWLs, how viewers respond to them, or whether a voluntary or mandated policy receives greater public support, studies involving representative samples of populations would seem appropriate. However, if the goal is to explore how specific sectors or high-risk groups respond to AWLs, then case studies or focus group designs would seem appropriate, since it might be challenging for some groups to have sufficient cases through general population-level sampling. Exploration of the awareness of the existence of AWLs, or the perception of the harms and risks of alcohol consumption, might use various methods, including randomized controlled trials and general population-level surveys. Understanding the features of a potential AWL that has not yet been implemented can be explored by on-line surveys, and on-line or in-person experiments. Ideally for this topic, the respondents should roughly represent major sectors of the eventual target population by age, ethnicity, sex/gender, and drinking practices. Questions about sponsorship by the alcohol industry, as well as broader questions about how policy-makers view AWLs or how policy-making evolves on AWLs, would benefit from the use of archival data, records of committee deliberations and key-informant interviews. If a new or substantially revised AWL is planned for a jurisdiction, then a quasi-experimental design is recommended, which includes several components, a before-and-after survey of persons expected to be exposed to the label, key informant interviews of patrons who used the retailers where the labelled bottles were sold, documentation of various responses to the new AWL, and official statistics on trends in alcohol sales in the intervention and comparison sites.

Although it is beyond the scope of this review, it is note-worthy that in a few cases (e.g., Ci et al. [158]; Roberts et al. [159]), warning signs in bars and retail alcohol outlets were linked with official statistics, such as birthweight of newborns. Ci et al. [158] examined point-of-sale alcohol warning signs (AWS), finding that AWS laws were associated with a decrease in the odds of alcohol consumption among pregnant women. Furthermore, the odds of very low birthweight and very pre-term birth decreased with AWS laws. Roberts et al. [159] found that mandatory state-level warning signs on alcohol were associated with lower odds of binge drinking, but not a decline in overall drinking.

The population foci of this body of research are skewed. A number of studies focused on adolescents and young adults, with the latter being accessible respondents for experiments or surveys conducted in university settings. Hankin et al. [108,109,110,111,112,113,114] reported on a number of studies involving African-American women who had given birth. However, for the most part race and ethnicity is not a central focus. Furthermore, there is need for more rigorous research on which messaging and type of format would have an impact on the high risk, binge drinkers, or those dependent on alcohol.

Three main alcohol-related harms were the main foci in these studies: pregnancy and alcohol, drinking and driving, and, more recently, alcohol-related cancer. In light of the extensive harm from alcohol, including numerous conditions, contexts and diseases [36,160], a somewhat broader range of message should be considered. A combination of better-known risks and not so well-known risks (cancer) seem like very good candidates for rotating or concurrent labels. Future work should consider including messages about addiction, liver disease, alcohol and diabetes, violence and mental illness.

From this literature we know relatively little on what policy-makers’ inclinations and preferences are. However, Neufeld et al. [12] point to the dynamics of compromise in wording in labels in CIS countries.

The alcohol industry, by design or default, appears to take a three-pronged approach to this topic: seek to postpone or block implementation of mandated warning labels [152]; promote voluntary messages that are vague or confusing [148]; or threaten legal action when they do not like the message, i.e., alcohol-related cancer [4]. With regard to the quasi-experiment in Yukon, they claimed:

- That Yukon had no authority to place warning labels;
- The study violated manufacturers’ constitutional rights to freedom of expression;
- That the messages were false and misleading, and hence defamatory.

None of these claims were considered to have any legal merit [124].

Considering warning messages on alcohol and cancer, Petticrew et al. [161] identified three main strategies: deny, omit or dispute the evidence that alcohol consumption increases cancer risk; mention cancer, but misrepresent the risk; and orient the discussion away from the independent effects of alcohol on common cancers.

Furthermore, and more broadly, Gleeson and O’Brien [154] examined alcohol labelling rules in free trade agreements. They concluded that the best legal interpretation of Trans-Pacific Partnership Agreement (TPP) supplementary labelling rules indicates that they do not impede the rights of governments to mandate warnings and other health information on alcoholic beverages, including the content of the messages, the inclusion of graphics, their colors and font, and their size and position on the container.

The alcohol industry is not enthusiastic about putting robust labels on their containers; indeed, they sometimes try to block such initiatives [161] or offer benign messages such as “please drink responsibly”. With regard to the latter, we did not find any evidence that this message reduced alcohol use among vulnerable groups, such as underage customers, or more generally reduced high-risk drinking.

What can be done to promote the uptake of robust AWLs? We offer three suggestions that are complimentary and inter-related. First, clear messages need to be conveyed to policy-makers, focusing on why AWLs are important, that there is public support for them, and that they are central components of the evidence-based alcohol warning label strategy (see Section 5 below). Furthermore, the messages need to debunk myths, including the myth that AWLs are too complicated or costly for the industry to implement, that it is illegal to put AWLs on alcohol containers, and that the evidence of the alcohol–cancer link is suspect.

Alcohol labels are changed periodically by the alcohol industry to herald various special occasions, such as holidays and sporting events, despite costs. It follows that the alcohol industry should be persuaded to regularly change their labels to promote health and reduce harm. Engaging decision-makers in this initiative to mandate robust labels with a range of different alcohol-related risks could take various forms, such as letters to political decision-makers, briefing notes, webinars, and presentations to government committees.

A second option is to seek legal action. There is a “duty of care” with regard to the products that manufacturers and governments approve for sale and distribution. As noted by Robert Solomon, referring to the industry response to the Yukon project, “All Canadian manufactures and suppliers have a duty to inform consumers of the risk inherent in the foreseeable use and misuse of their products” [162]. Furthermore, as noted by Kruger [13] prior to the US label being implemented: “Alcohol beverage manufacturers should be required to provide adequate warning to consumers”.

Third, stake-holders could be engaged in order to build support for government mandated effective AWLs. Since there are many harms related to alcohol use, it seems feasible that this informal network to support such initiatives could include: oncologists, FASD specialists, those involved in preventing alcohol-related chronic disease or alcohol-related violence, and those involved in alcohol-related driving counter-measures. Various NGOs can also play an important role in countering industry attempts to block mandating AWLs. 

The 1989 US legislation on alcohol warning labels stimulated dozens of publications that were directly and indirectly linked to this mandate. As noted in the previous sections, and the review by Stockwell [35], there were a number of unique findings from assessments of the US AWL. 

That there were any substantial impacts from the US warning labels is surprising, since the labels, through various compromises, appear to have been designed for modest impact. The font is small, often a color similar to the background, with no graphics or striking layout and no rotating messages, and labels are often placed on the back of the bottle or can.

Some 30 years later, the Yukon quasi-experiment illustrated what is feasible with a robust label and attendant research design. This study provides an exemplary model for evaluation of new alcohol labeling initiatives in other settings.

Several authors have advocated for alcohol labelling as part of a larger package of interventions; for example, Wilkinson and Room [14] and Neufeld et al. [12]. In the WHO European Region Report on alcohol labelling practices [39], the authors point to Israel, Lithuania and Ireland, which had introduced AWLs as part of broader prevention strategies focusing on alcohol.

However, most warning label studies, especially those focusing on populations, did not document what other alcohol policies were in place or underway in the jurisdiction during the period of evaluation of AWLs. Therefore, future studies should include designs where an introduction of an alcohol warning label is part of other alcohol policy initiatives, such as alcohol pricing, physical availability, or controls on marketing, for example. It would be advantageous if the implementation of change was in stages and research design allowed for assessing both the independent and combined impact of the other policy components and the alcohol warning labels.

## 5. Recommendations

Alcohol consumption contributes to a wide range of chronic, acute and social problems [163]; these problems not only impact the drinkers but others, including innocent victims. Alcohol warning labels are an important component of a comprehensive and evidence-based policy response needed to reduce alcohol-related injury, disease and death [2,3,36,39]. As shown in Section 3 above, they can increase awareness of risks, change drinking behavior and reinforce a perspective that alcohol is not an ordinary commodity, and requires special attention and regulation. Furthermore, there is substantial public support for alcohol warning labels in surveys conducted in several countries [137,144].

The following recommendations draw on several resources: those offered recently by Kokole et al. [36], the WHO European Office report by Jané-Llopis et al., [39], the WHO snapshot report on health warning labels on alcoholic beverages, previous reviews [14,16,20,28], the recommendations offered in a number of publications included in our analysis [45,51,61,66,97,129,147,164,165], and a document developed by the Canadian Alcohol Policy Evaluation Project Team [166]:

1. Alcohol warning labels should be an essential component of regional, national and international alcohol policies that include evidence-based interventions and other effective prevention strategies, and should effectively warn customers [13,39,74,75,156].
2. When implementing alcohol warning labels, steps should be taken to implement them in combination with other avenues of prevention messaging and to assess their impact in combination with other alcohol policy actions [6,28,36,39,45,105,120,127].
3. So far, the evaluations and experimental studies have been based on relatively few sites. Countries that are planning to implement AWLs should actively seek ways to evaluate and frequently monitor their impacts on knowledge, awareness of alcohol issues and risks, conversations about alcohol, intentions to change drinking behaviors, and actual changes in drinking behavior [6,8,106].
4. New initiatives involving alcohol warning labels should build on the best evidence, including the extensive research of the 1989 US alcohol warning label legislation [66,69,99,103,136], the more recent exemplary research of the Yukon experiment [8,106,107,133], and studies conducted in Australia [77,129,147], the United Kingdom [61,126,151,164] and elsewhere, and several reviews and guideline documents [6,36,39,166].
5. A combination of methods is recommended to assess their impact on knowledge of the harms and risks of alcohol consumption, perception of risk, conversations about alcohol-related risks, and drinking behavior. These could include surveys, focus groups, key informant interviews, archival data on alcohol sales and drinking behavior, and use of a quasi-experimental design [8,36,106,107].
6. These new AWLs should be government-mandated, ensure that enforcement mechanisms are in place, and their implementation regularly monitored and documented [6,12,25,36,39,81,97,145,147,148,150,151,166].
7. Innovative designs should be used, including a combination of text and images, large font, bright colors and placement on the front of cans and bottles containing alcohol [6,25,28,36,39,44,47,50,61,66,78,164,165].
8. The messages should be clear, factual and specific, and not include vague and confusing phrases, such as “please drink responsibly” [6,8,36,39,49,55,66,67,84,88,166].
9. The messages should refer to several alcohol-related risks, including, for example, several types of cancer, liver disease, risk of injury, alcohol-related violence, drinking and driving, and harm to the fetus [6,28,35,36,87,93,106,107,126,128,134,166].
10. In order to avoid clutter and small font, and to facilitate enhanced attention, one preferred option is having several different messages be rotated on a range of alcohol products [14,36,43,166].
11. The labels should also include standard drink information and link to the low-risk drinking guidelines used in the jurisdiction [6,7,12,15,28,29,35,60,67,68,94,107,166].
12. Tracking, monitoring and assessment of the impacts of alcohol warning labels should focus on several sectors, including: the general population, adolescents and youth, women of child-bearing age, high-risk drinkers, and those dependent on alcohol [6].

## 6. Conclusions

Awareness of the existence of AWLs tends to be greater among certain groups, such as drinking drivers, impaired drivers, drinkers, pregnant women and those of younger age. The AWLs sponsored or prompted by the alcohol industry tended to have limited efficacy, were small in size, voluntary, and had low recall. In some cases, the intent of the messages was confusing to respondents. Studies on design and message content point to expected greater impact from a combination of large size and easily noticeable messages that include text and graphics and refer to lesser-known alcohol-related risks such as cancer. Recent high-quality research indicates an impact from AWLs on drinking behavior. The evidence in support of AWLs is growing and points to design and messaging features to enhance potential impact. AWLs are an important component of multi-dimensional country level and sub-national alcohol control strategies.

## Figures and Tables

**Table 1:** Search Strategy.

**Embase, MEDLINE**	Query (‘alcohol’/exp OR alcohol) AND (health AND warning OR ‘health warning’ OR (front AND of AND package) OR ‘front of package’ OR (message AND on AND harms) OR (warning AND label)) Mapped terms n/a
**Psycinfo**	((AnyField:(health warning)) OR (AnyField:(warning label)) OR (AnyField:(front of package)) OR (AnyField:(message on harms)))
**Pubmed**	(“ethanol”[MeSH Terms] OR “ethanol”[All Fields] OR “alcohol”[All Fields] OR “alcohols”[MeSH Terms] OR “alcohols”[All Fields]) AND ((“health warning”[All Fields] OR “warning label”[All Fields]) OR “front of package”[All Fields])
**Scopus**	TITLE-ABS-KEY (alcohol AND (health AND warning OR label OR front-of-package OR advertisem)

## Data Availability

The resources are available in the citations and Supplementary Tables.

## Supplementary Materials

The following are available online at https://www.mdpi.com/article/10.3390/ijerph191811676/s1 [167,168,169,170,171,172].
