# Supplementary material for: Alcohol Health Warning Labels: A Rapid Review with Action Recommendations
Source: Int J Environ Res Public Health. 2022 Sep 16;19(18):11676. doi: 10.3390/ijerph191811676 (PMC9517222; doi:10.3390/ijerph191811676)
Supplement: Supplementary file 1 [file ijerph-19-11676-s001.zip › S Table S4 Adolescents revised. August 24.pdf]

Supplementary Table S4: Adolescents and Alcohol Warning Labels

**Author & year**	**Goal**	**Design & source of data**	**Impact/outcome**
MacKinnon et al 1993 [1]	US. Determine awareness of the alcohol labelling law, exposure to the warning label,' and beliefs about and memory for the risks listed on the label	Before and after study - pre- and post AWL legislation, 1989, 1990. Before 1,211 grade 12 students. After 2,006 grade 12 students.	After the AWLs appeared, more adolescents had seen it, were aware that it existed, and were able to recognize the risks written on the label. No positive change in beliefs about the risks on the label was observed.
Nohre et al, 1999 [2]	US. Examine the association between receiver characteristics and awareness of, exposure to, memory for, and beliefs about AWLs	Pre and post AWLs appearing, 1989, 1992. Independent cross-sectional samples (n = 6,393) grade 12	Students who were from lower SES, had lower usual grades, and were heavier drinkers were more aware of the warning label law and had more exposure to the warning. Non-White students were more aware of the warning law but did not report more exposure. Students who drank from the alcohol container (can or bottle) and students with more friends who drank alcohol had more exposure to the warning. Students who were female, White, heavier drinkers, had more friends who drank alcohol, and drank from the alcohol container had more accurate memory for the risks on the warning. Students who were female, had higher grades, attended religious services more frequently, watched less television, and drank less alcohol were more likely to believe the risks on the warning.
MacKinnon et al, 2000 [3]	US. Examine the effects of the alcohol warning label on adolescents during the first 5 years that the warning was required.	Surveys were administered to 10th-grade (n = 16,661) and 12th grade (n = 15,856) students from the 1989–1990 school year through the 1994–1995 school year.	There were increases in warning awareness, exposure, and recognition memory. These effects leveled off approximately 3.5 years after the inclusion of the warning on alcohol beverage containers. There was no beneficial change attributable to the warning in beliefs, alcohol consumption, or driving after drinking.
Moss et al, 2017 [4]	UK. To examine the effects of exposure to health messages on attitudes towards drinking and drunkenness, and intentions to drink	Pre and post – 94 participants were either actively or passively exposed to one of two health messages (Responsible drinking messages]RDM] or general wellbeing).	Active exposure to RDMs led to more positive attitude towards drunkenness, while passive exposure led to more negative attitudes. Passive RDM exposure led to increased intentions to get drunk in future. Wellbeing posters produced the opposite effect in some but not all of these measures. Exposure to RDMs may have some beneficial effects in terms of creating more negative attitudes towards alcohol consumption, but we also identified potential iatrogenic effects regarding attitudes and intentions towards drunkenness amongst an underage sample of drinkers

	and get drunk, amongst underage drinkers.		
Critchlow et al, 2019 [5]	UK. Examine awareness and recall of such information and messaging among adolescents in the United Kingdom	A cross-sectional survey was conducted in 2017 with 11–19 year olds (n = 3,399), participants asked if they had seen any information, health messages or warnings on alcohol packaging in the past month (Yes/No) and, if so, what they recalled.	About 1/3 recalled seeing product-related information, health messaging or warnings on alcohol packaging in the past month/<br>Awarness was greater among current drinkers than non-drinkers, high-risk drinkers versus lower-risk drinkers, and susceptible never-drinkers than non-susceptible never-drinkers. Ten messages were recalled, with drinking responsibly (18%) and not drinking during pregnancy (13%) most recalled
Morgenstern et al, 2021 [6]	Germany. Present AWLs to students	Health survey, 2018. N = 9,260 students age 10-17	Exposure to an AWL affected alcohol-related cognitions of children and adolescents. This was true for both, text based or picture-and-text-based labels. Pictorial messages seem to be more potent to elicit emotions, at least for recipients that already have experience with alcohol use.

References

1. MacKinnon DP, Pentz MA, Stacy AW. The alcohol warning label and adolescents: The first year. Am J Public Health [Internet]. 1993;83(4):585–7. Available from: https://www.embase.com/search/results?subaction=viewrecord&id=L23109844&from=export

2. Nohre L, MacKinnon DP, Stacy AW, Pentz MA. The association between adolescents' receiver characteristics and exposure to the alcohol warning label. Psychol \& Mark. 1999 May;16(3):245–59.

3. MacKinnon DP, Nohre L, Pentz MA, Stacy AW. The alcohol warning and adolescents: 5-year effects. Am J Public Health. 2000 Oct;90(10):1589–94.

4. Moss AC, Evans S, Albery IP. Effect of Health Messages on Alcohol Attitudes and Intentions in a Sample of 16-17-Year-Old Underage Drinkers. Int J Environ Res Public Health. 2017 Oct;14(10).

5. Critchlow N, MacKintosh AM, Thomas C, Hooper L, Vohra J. Awareness of alcohol marketing, ownership of alcohol branded merchandise, and the association with alcohol consumption, higher-risk drinking, and drinking susceptibility in adolescents and young adults: a cross-sectional survey in the UK. BMJ Open. 2019 Mar;9(3):e025297.

6. Morgenstern M, Dumbili EW, Hansen J, Hanewinkel R. Effects of alcohol warning labels on alcohol-related cognitions among German adolescents: A factorial experiment. Addict Behav [Internet]. 2021;117. Available from: https://www.embase.com/search/results?subaction=viewrecord&id=L2011051022&from=export
