# Supplementary material for: Alcohol Health Warning Labels: A Rapid Review with Action Recommendations
Source: Int J Environ Res Public Health. 2022 Sep 16;19(18):11676. doi: 10.3390/ijerph191811676 (PMC9517222; doi:10.3390/ijerph191811676)
Supplement: Supplementary file 1 [file ijerph-19-11676-s001.zip › S Table S7 Pregnancy. revised. Aug. 24. docx.pdf]

Supplementary Table S7: Pregnancy and Alcohol Warning Labels

Author & Year	Location & Goal	Design & source of data	Impacts/outcomes
Mayer et al. 1991 [1]	US. Evaluate the preliminary impact of a newly required warning label for alcoholic beverage containers.	Observational of 400 Utah households. April 1989	The warnings have achieved a considerable level of public awareness, with the admonition against drinking during pregnancy being the most memorable portion of the warning. But there is no evidence yet that the labels have affected knowledge of the health and safety risks associated with alcohol or self-reported alcohol consumption.
Mazis et al. 1991 [2]	US. The impact of alcoholic beverage warning labels	National surveys May 1989 n=1,008 May 1990 n=1,020	A slight increase in the public's perception of the risk level associated with consuming alcoholic beverages since the warning label became effective. About 11 percent of the sample was able to identify the specific pregnancy hazard warning. The awareness level was highest among younger adults and "heavy" alcohol consumers.
Barrett et al. 1993 [3]	Illinois. To examine (1) the prevalence of self-reported alcohol use among women of childbearing and (2) their ability to recall information about pregnancy risk contained in alcohol warning labels and signs.	Telephone survey.Summer 1990 RRD n=1,515	There were no significant differences between pregnant and nonpregnant women in self-reported alcohol use in the past year. More than one fourth (28.3%) of all women were able to recall information about pregnancy risk information contained in warning labels on alcoholic beverage containers, Almost three times as many pregnant women (22%) as nonpregnant women (8.3%) were able to recall information about pregnancy risk contained in alcohol warning signs (x2[l]=6.3, P<.05).
Hankin et al. 1993 [4]	Huzel Hospital prenatal clinic, Detroit. Tested the hypothesis that the Federal Beverage Labelling Act of 1988 has	Time series analysis of 12,026 consecutive African American inner city women at prenatal clinic,	Results of time series analysis indicated a 7-month lag in the impact of the alcohol warning label. Controlling for population changes, antenatal drinking began to fall as of June 1990. However, this decrease was small in size and did not impact on the heaviest drinkers.. Preliminary analysis showed that while periconceptional drinking rose, antenatal drinking remained constant over this period.

	decreased antenatal drinking	Sept. 1986 to Sept. 1991	
Hankin et al. 1993 [5]	Detroit. Whether drinking by pregnant women has decreased since the AWLs in US,	Qualitative. Sample of 4,379 consecutive black pregnant women. May 22, 1989 to Sept. 30, 1991	Four-month lag between implementation of label law (Nov. 1989) and significant increase in women's awareness of a warning label (March1990). At the time of conception, 44% of the women were not drinking, 42% were lighter drinkers (consuming less than .5 ounce of absolute alcohol per day), and 14% were risk drinkers (consuming .5 ounce or more of absolute alcohol per day). At the time of entry into prenatal care, the average woman consumed .05 ounce of absolute alcohol per day. 37% had seen AWLs Nov. 18/89 to May 30/90; 56% had seen between June 1/90 to Sept. 30/91.Neither the post-label variable nor awareness of the label are significant predictors of in-pregnancy drinking.
Hankin et al. 1993 [6]	Inner city Detroit. The penetration of the label on store shelves and to ascertain whether the labeling varied by the type of alcoholic beverage,	Consecutive African-American gravidas n=5,169 at Hutzel prenatal clinic. May – June 1990	During the time period before the warning label law went into effect (May 22, 1989-November 17, 1989) between 3 1 % and 41 % of the women said a warning label existed. After November 17, 1989, the percentage aware ranged from 3 1 % to 75 % of the gravidas, with the level of awareness increasing as time passed.The one-time survey of retail outlets conducted 7 months after the implementation of the law revealed that while 86% of beer and 69% of wine coolers were labeled, only 34% of wine and 30% of liquor containers were labeled in June 1990. Women under 30 years old were 1.55 times more likely to know about the warning label than older women. Results of the time series analysis and logistic regression: (1) there was a high level of false-positive awareness; (2) a significant increase in awareness began in March 1990; (3) women consuming beverages with shorter shelf-lives had higher levels of awareness; and (4) younger women were more aware of the label.
Hankin et al. 1994 [7]	To examine the impact of the AWL on the attitudes and drinking behavior.	Natural experiment. Time series analysis. African American pregnant women n=3,572	Knowledge of the Warning Label increased 3 months after the implementation of the law. Drinking during pregnancy declined after a 7-month lag only among non-risk drinkers, although birthweight showed no trends. Decline in drinking was small, about equivalent of one once of beer per week.
Hankin et al. 1996 [8]	Detroit. To examine whether awareness of the warning label has reached its upper limit. In addition, the predictors of awareness were analyzed.	Sample of 7,334 of African American women seeking care at Hutzel prenatal clinic. May 22, 1989 through June 30, 1993.	There was a substantial false-positive rate: between 29% and 41% said a warning label existed. After November 17, 1989, the level of awareness increased over time, from 29% to 78%.The maximum level of label awareness in this population is -80%, and knowledge of the warning label has not changed substantially from May 1992 to June 1993.<br>Older women were less likely to know about the label. Risk drinkers who would have more frequent exposure to the warning label had higher levels of awareness.

Supplementary Table S7: Pregnancy and Alcohol Warning Labels

Hankin et al. 1996 [8]	Detroit. The impact of the Federal AWL on multiparae (women with at least one previous live birth) and nulliparae (women with no previous live births).	Time series analysis to compare the pre- and post-labelled drinking of multips and nullips. N=17,456 inner city black graidas. September 1986 to September 1993	For nulliparea n=7,349 reported drinking showed a significant decline in June 1990, 7 months after implementation of AWL. In contrast, multiaparae n=10,107 showed no change in reported drinking postlabel.<br>Multips were likely not less aware of label - which may suggest denial process. Multips drank beverages more likely to be labelled at the time so likely more exposed to AWL. Multips may have ignored WL because based on prior experience, thought alcohol will not harm their fetus
Hankin et al. 1998 [9]	Detroit. Study examined drinking during pregnancy before the AWL was implemented and afterwards.	Time series analysis N=21,127 African American pregnant women. 1986-1995	Short-lived decline in in-pregnancy drinking score about 8 months after AWL introduced, as unemployment declined, and drinking increased as unemployment increased
Chambers et al. 2005 [10]	San Diego County. The prevalence of alcohol consumption in a sample of low-income pregnant Latinas and examined risk factors for alcohol use in the periconceptional period	Cross-sectional survey in-person in home interview. 100 pregnant Latinas from 2,749 women. 2001-2003	More women who reported any drinking during the periconceptional period had heard of FAS and could correctly identify outcomes associated with prenatal alcohol exposure compared to non-drinkers<br>Significantly more women who reported any amount of alcohol as well as binge drinkers recalled having seen warning messages on cans or bottles, and binge drinkers were also more likely to have seen warning signs in public places.
Blume and Resor, 2007 [11]	US. To investigate the knowledge of Mexican origin women living in colonias about potential risks related to drinking behaviour	Survey of 99 Hispanic women age 12-67. 70 reported being pregnant at least once	English language skills significantly predicted participants' ability to remember health warnings on beverage containers whereas greater awareness of nutritional information on labels was associated with lesser amounts of alcohol consumed. Beliefs that drinking during pregnancy is helpful and not associated with liver and cognitive problems were significantly associated with higher alcohol consumption, and beliefs that drinking helps when pregnant along with a reported history of drinking during a previous pregnancy significantly predicted self-reported drinking during a most recent pregnancy.

Supplementary Table S7: Pregnancy and Alcohol Warning Labels

Parackal et al. 2010 [12]	New Zealand. To report the rating of a national sample of 16–40-year-old non pregnant New Zealand women on AWLs as a source of information on risks associated with alcohol consumption in pregnancy.	N=1,129 age 16-40	A response rate of 65%. Just over half of the women surveyed gave a high rating for a warning label as a source of information on alcohol consumption in pregnancy. Women below 30 years of age and who were of non-European ethnicity were more likely to give a high rating compared with older women and European women, respectively
Bazzo et al. 2012 [13]	Italy. To assess the impact of the image on the target population 1 year after the launch of the campaign.	Semi-structured self-reported questionnaire n=694. To parents or care-givers of children age 0-2 coming to vaccination clinic. May 2010	Overall, 84.3% (n = 582) of the respondents said that they remembered the image.The image generally seemed to arouse a high impact on the majority of the sample: ~38% indicated distress and 40% liking. Almost all respondents recalled the warning message conveyed by the image ('if a pregnant woman drinks alcoholic beverages, it can harm the baby'). About half of the sample (53.3%, n = 368) said that the image suggested at least one of the actions expected, while 32.9% (n = 227) reported only generic assertions (e.g. 'I think about the possible side effects of alcohol') or not expected actions (e.g. 'do not drink during the whole life span' or 'do not drink if you are young') and 13.8% (n = 95) did not respond. Half of the sample recalled the health behaviours suggested by the campaign ('do not drink in pregnancy' and 'spread the health message').
Dumas et al, 2018 [14]	France. The aim of this study was to evaluate current perceptions of risks and awareness of the official recommendations regarding drinking during pregnancy and during breastfeeding	Quota sampling, of cross-sectional study, based on telephone interviews of a representative sample of pregnant and postpartum French women n=3,603. May – July 2012.	92% thought drinking 1-2 alcoholic drinks daily during pregnancy could be harmful to the unborn child; proportion was higher among drinkers than non-drinkers. 86% thought only one binge drnking episode during pregnancy could be harmful to the foetus. 41% believed that spirits were more harmful than wine or beer to the unborn child. 9% thought that drinking beer while breastfeeding was recommended.66% of women had noticed the warning label on alcohol containers.Of those who had seen it, 99% thought it suggested abstinence. Unawareness of AWL higher in women age 35 or over than in women age 25 or less. Thus, the French warning label may not be effective in promoting complete abstinence because understanding of the concept of a 'standard drink' remains low.
Odeigah et al. 2020 [15]	Nigeria & UK. This study compared volume, alcohol by	The information on the product labels of 59 selected beers and spirit	Pregnancy and drink driving warnings were present on 18.2% of product labels of alcoholic beverages produced in Nigeria. Product labels on alcoholic beverage brands produced in the UK were more likely than brands produced in Nigeria to include a 'don't drink when pregnant' logo (100% vs. 18.2%). Health warnings were less common in brands in Nigeria than UK.

	volume (ABV), alcohol units, and health warnings on product labels of selected alcoholic beverages simultaneously produced in Nigeria and the UK	drinks produced in Nigeria was assessed based on six mandatory elements: Under age population, pregnant women, general population.<br>13 alcoholic beverages produced in Nigeria and UK	

References

1. MAYER RN, SMITH KR, SCAMMON DL. EVALUATING THE IMPACT OF ALCOHOL WARNING LABELS. In: HOLMAN, RH and SOLOMON, MR, editor. ADVANCES IN CONSUMER RESEARCH, VOL 18. PROVO: ASSOC CONSUMER RESEARCH; 1991. p. 706–14. (ADVANCES IN CONSUMER RESEARCH; vol. 18).
2. MAZIS MB, MORRIS LA, SWASY JL. AN EVALUATION OF THE ALCOHOL WARNING LABEL - INITIAL SURVEY RESULTS. J PUBLIC POLICY \& Mark. 1991;10(1):229–41.
3. Barrett ME, Wong FY, McKay DR. Self-reported alcohol use among women of childbearing age and their knowledge of alcohol warning labels and signs. Arch Fam Med [Internet]. 1993;2(12):1260–4. Available from: https://www.embase.com/search/results?subaction=viewrecord&id=L24874420&from=export
4. Hankin JR, Firestone IJ, Sloan JJ, Ager JW, Goodman AC, Sokol RJ, et al. . The impact of the alcohol warning label on drinking during pregnancy. J PUBLIC POLICY \& Mark. 1993;12(1):10–8.
5. Hankin JR, Sloan JJ, Firestone IJ, Ager JW, Sokol RJ, Martier SS, et al. The alcohol beverage warning label - when did knowledge increase. Alcohol Exp Res. 1993 Apr;17(2):428–30.
6. Hankin JR, Sloan JJ, Firestone IJ, Ager JW, Sokol RJ, Martier SS. A time-series analysis of the impact of the alcohol warning label on antenatal drinking. Alcohol Exp Res. 1993 Apr;17(2):284–9.
7. Hankin JR, Firestone IJ, Sloan JJ, Ager JW, Sokol RJ, Martier SS. Time series analyses reveal differential impact of the Alcohol Warning Label by drinking level. Appl Behav Sci Rev [Internet]. 1994;2(1):47–59. Available from: https://www.scopus.com/inward/record.uri?eid=2-s2.0-

34247716519&doi=10.1016%2F1068-8595%2894%2990020-5&partnerID=40&md5=a6f06235500c168a15bf666ac3cb037b

8. Hankin JR, Firestone IJ, Sloan JJ, Ager JW, Sokol RJ, Martier SS. Heeding the alcoholic beverage warning label during pregnancy: Multiparae versus nulliparae. J Stud Alcohol. 1996 Mar;57(2):171–7.

9. Hankin JR, Sloan JJ, Sokol RJ. The modest impact of the alcohol beverage warning label on drinking during pregnancy among a sample of African-American women. J PUBLIC POLICY \& Mark. 1998;17(1):61–9.

10. Chambers CD, Hughes S, Meltzer SB, Wahlgren D, Kassem N, Larson S, et al. Alcohol consumption among low-income pregnant Latinas. Alcohol Exp Res. 2005 Nov;29(11):2022–8.

11. Blume AW, Resor MR. Knowledge about health risks and drinking behavior among Hispanic women who are or have been of childbearing age. Addict Behav [Internet]. 2007;32(10):2335–9. Available from: https://www.embase.com/search/results?subaction=viewrecord&id=L47198955&from=export

12. Parackal SM, Parackal MK, Harraway JA. Warning labels on alcohol containers as a source of information on alcohol consumption in pregnancy among New Zealand women. Int J DRUG POLICY. 2010 Jul;21(4):302–5.

13. Bazzo S, Battistella G, Riscica P, Moino G, Marini F, Geromel M, et al. Evaluation of the Impact of the Image Used in a Communication Campaign to Raise Awareness about the Effects of Alcohol Use During Pregnancy(dagger). ALCOHOL Alcohol. 2012;47(6):657–62.

14. Dumas A, Toutain S, Hill C, Simmat-Durand L. Warning about drinking during pregnancy: Lessons from the French experience. Reprod Health [Internet]. 2018;15(1). Available from: https://www.embase.com/search/results?subaction=viewrecord&id=L620433604&from=export

15. Odeigah OW, Olley BO, Patton R, Dumbili EW. Fitting a quart in a pint pot: a comparison of selected alcoholic drinks produced in Nigeria and the UK. Drugs Educ Prev Policy [Internet]. 2020; Available from: https://www.scopus.com/inward/record.uri?eid=2-s2.0-85083566096&doi=10.1080%2F09687637.2020.1740652&partnerID=40&md5=0ad56c8a5b7908eb0fcaae8a937d703b
