# Supplementary material for: Alcohol Health Warning Labels: A Rapid Review with Action Recommendations
Source: Int J Environ Res Public Health. 2022 Sep 16;19(18):11676. doi: 10.3390/ijerph191811676 (PMC9517222; doi:10.3390/ijerph191811676)
Supplement: Supplementary file 1 [file ijerph-19-11676-s001.zip › S. Table S1 Design. revisd. Aug 24.pdf]

Supplementary Table S1. Design of Alcohol Warning Labels

Author & Year	Location & Goal	Design & source of data	Impacts/outcome
Godfrey et all. 1991 [1]	U. of Houston. Which factors affect the noticeability of the mandated alcohol warning labels	Experiment. 60 subjects were presented with either a alcohol container with or without a warning.	More likely to miss WL when on neck than front.Vertical more likely to be missed than those printed horizontal. Significanlty faster in identifying warnings printed on the front, Government warning phrase helped shorted detection time. More noticeable warnings associated with less "clutter" around the AWL.
Gorn et al. 1996 [2]	U. of British Columbia University students create warning labels which focus on the issue of DUI	Design a label aimed at student of the same sex and designed at students of the opposite sex. 55 undergrads	The seven labels had several characteristics in common. They all contained a personal reference to "you" or "your." The consequences of drinking and driving were realistic, relevant and evoked anxiety.
Al-hamdani and Smith 2015 [3]	Canada. Alcohol packaging options and test whether labelling alters consumer perceptions	RTC with 4 labelling conditions 92 adult respondents	Participants perceived bottles with warnings less positively as compared to standard bottles in terms of product-based and consumer based perceptions. Plain bottles showed the most consistent statistically significant results, followed by text and image warnings, and then text warnings in pair-wise comparisons with the standard bottles. Some support for the impact of plain packaging on warning recognition was also found;
Monk et al. 2017 [4]	UK. To examine attention levels to different types or alcohol warning labels	Eye-tracking study. 22 participants viewed neutral or graphic warning messages. Pre and postexposure outcome expectancies were assessed.	Although attention was heightened for images (as opposed to the text within the warnings), attention levels did not vary between graphic and neutral imagery. Positive outcome expectancies were associated with individuals attending to the imagery for longer time periods.
Wigg et al. 2016 [5]	UK. Effectiveness of health warning labels on alcoholic beverages	Participants presented with one of three health warnings: none, text only, pictorial, and responded to fear arousal and perceptions toward alcohol use. 60 college students	Pictorial health warnings were associated with significantly higher fear arousal, increased perceptions of the health risks of consuming alcohol as well as greater intentions to reduce and quit alcohol consumption compared to the control.

Supplementary Table S1. Design of Alcohol Warning Labels

Zahra et al. 2015 [6]	Uk. To investigate the cognitive processing of emotive pictorial warnings.	Study 1 (n=153), evaluated warnings in which content type (Alcohol and Non-Alcohol) and emotional valence (Positive and Negative) were manipulated through imagery. Study 2 (n=58), participants evaluated the certainty of outcomes described by alcohol-related and non-alcohol-related warnings.	Study 1 found that in alcohol-related warnings, there was no difference in reasoning accuracy between positive and negative content. However, fewer correct responses followed exposure to negative general-health messages. Study 2 suggested that when a warning involves the potential consequences of drinking alcohol, accuracy is improved when the content is negative. However, when considering the consequences of abstinence, accuracy was greatest when the content was positive.
Annunziata et al. 2016b [7]	Italy. Interests and preferences of Italian consumers toward AWL information.	On-line survey, 300 over age 18	Majority of Italian wine consumers are interested on health warnings on the wine label and tend to prefer a logo plus the claim. Consumers consider particularly useful the warning "do not drive after drinking" and "avoid drinking alcohol a when you are taking medicines".
Annunziata et al. 2016a [8]	Four regions in Italy. What would consumers like in see in nutritional and health warnings on wine labels?	Survey online Qs<br>Co-joint analysis, age 18-75	Cluster 2 (health warning seekers) is the largest of those identified, accounting for 48% of respondents. Respondents in this group attach more value to the presence of a health warning, preferring the version with a logo (30%), followed by nutritional information, preferring the picture card with the glass indicating the amount of kcal (25%) and price (24%). This group also has the lowest level of wine involvement compared with the other clusters.
Annunziata et al. 2016c [9]	France, Italy and Spain, US<br>Insights into consumer support, interests and preferences.	On-line survey. 330 in Italy, 185 France, 195 Spain, 306 east coast USA. Age 35-54	Respondents were presented with five different warning statements and asked to express how useful they consider such statements in their choices. Consumers from all countries tended to assign a high utility score to "ban on alcoholic beverages to children under 18/21 years" and "do not drive after drinking" ."Avoid drinking alcohol during pregnancy" was considered, on average, more useful by US and French consumers. Moreover, for the warning "avoid drinking alcohol when you are taking medicines", was of higher utility of American consumers.
Coomber et al. 2017a [10]	Melbourne. Assessed graphic warnings which included the use of	Focus group U students age 18-25. 16 female 11 male	Pictorial and graphic warnings stand out to young adult drinkers, convey new information, and elicit a negative emotional response. However, they did not believe the warnings would deter them from risky drinking. While participants stated the warning size and placement ensured warnings stood out from the product label and were easily noticed, they specified a preference

	full color photographs (as opposed to drawn images) and the color red in the warning heading.		for such warnings to be placed on the back of the label. Over time, the warnings would simply become "white noise" and no longer serve their purpose
Pham et al.2018 [11]	Australia. Attention scores obtained through self-report methods are compared to objective measures (eye-tracking).	Multi-method experimental design four conditions in eye-tracking study; control , colour, size and colour, size. N= 449 in study 1 and 87 in study 2.	Eye-tracking identified that 60% of participants looked at the current in market alcohol warning label while 81% looked at the optimized design (larger and red). In line with observed attention self-reported attention increased for the optimized design.
Al-Hamdani, Smith 2017 [12]	Nova Scotia H1. Assess larger graphic warnings with bottles possessing medium graphic labels. H2. Assess plain packaged bottles vs. branded bottles.	On-line experiment. 18 conditions. Convenience sample 440 adults	Hypothesis 1 was partially supported—distilled spirit and wine bottles with extra large warnings received lower product-based ratings compared with their medium-sized warning counterparts. Hypothesis 2 was partially supported—the mean product based and consumer-based ratings were lower for plain packaged distilled spirits, wine, and beer bottles compared with their branded counterparts but boringness ratings were not higher
Dossou et al 2017 [13]	Rennes, France. Effectiveness of two mandatory warnings introduced in France in 1991 and 2007;	In-depth interviews were conducted with 26 French people aged 15–29 years.	While both warnings suffered from a lack of visibility and noticeability due to their size, location, and outdatedness and because of competition from marketing design elements, the warning on the advertisement that followed content regulations was most visible. For the majority of participants, recall and awareness were higher for the text warning on the advertisements than for the pictogram. While the pictogram was generally understood, the majority of participants lamented its lack of explicitness The majority of participants said that the alcohol warnings were not effective in terms of changing behaviour.
Authayarat et al. 2018 [14]	Thailand. To design and validate emotional warning pictorials for alcohol	Experiment. Selected 60 warning pictorials into three affective state groups, positive, neutral,	The result showed that affective warning pictorials were able to evoke specific affective state of warning pictorials to participants such as positive, neutral, and negative state. Affective warning pictorials from this research can be implemented as the alcohol container labels.

	container labels in order to increase awareness of risks from alcohol consumptions	and negative. Male respondents 18-44	
Sillero-Rejon et al. 2018 [15]	Spain. Explore how the size and design of alcohol by volume (ABV) labels, along with the alcohol strength presented on these labels, influence visual attention toward them.	Experimental human laboratory study with control group N=64 adults	Results showed strong evidence that the number of fixations was higher when the ABV labels were larger and used a traffic light system. Likewise, we found a higher number of fixations toward larger health warning labels and differences in visual attention depending on the ABV content presented.
Vasiljevic et al. 2018 [16]	UK. Whether labeling wine and beer as lower in alcohol increases their consumption.	RCT. Weekly wine and beer drinkers (n 264) sampled from a representative panel of the general population of England were randomized to one of three groups to taste test drinks in a bar-laboratory varying only in the label Displayed.	The total amount of drink consumed increased as the label on the drink denoted successively lower alcohol strength. Group contrasts showed significant differences between those offered drinks labelled as Super Low compared with Regular. There was no significant difference in amount consumed between those offered drinks labelled as Low compared with Regular
Annunziata et al. 2019 [17]	Italy & France. To analyse the interest, attitudes and preferences of Generation Y consumers3 (those born between 1978 and 2000) towards different	On-line survey. Two samples of complete responses. Discrete choice experiment. The design was composed with three alternatives.	Prefer no warning sign. Prefer warning on the back of bottle where it is less visible. Negative reaction to logo that warns of long term effects of wine on the brain. Neutrally framed messager preferred to those negatively framed. Italians willing to accept drinking and driving warning logos; French respondents negatively evaluated both messages (drinking and driving and brain damage) (France already has a mandatory warning logo on the back labels (a pregnant woman)

	formats of health warnings on wine labels.		
Gold et al. 2020 [18]	England.Tested the effect of different pictorial representations of alcohol content, some with a health warning, on knowledge of the UKs LRDG and understanding of how many drinks it equates to.	Parallel randomized controlled trial. On-line . **Interventions** The control group saw existing industry-standard labels; six intervention groups saw designs based on: food labels (serving or serving and container), pictographs (servings or containers), pie charts (servings) or risk gradients. A total of 500 participants (~70 per condition) saw a health warning under the design. answer).	In the control group, 21.5% knew the LRDG; proportions were higher in intervention groups (all *P*<0.001). The three best-performing designs had the LRDG in a separate statement, beneath the pictograph container: 51.1% [adjusted odds ratio (aOR) = 3.74, 95% confidence interval (CI) = 3.08–4.54], pictograph serving 48.8% (aOR = 4.11, 95% CI = 3.39–4.99) and pie-chart serving, 47.5% (aOR = 3.57, 95% CI = 2.93–4.34). Participants underestimated how many servings they could drink: control mean = _4.64, standard deviation (SD) = 3.43; intervention groups were more accurate (all *P* < 0.001), best performing was pictograph serving (mean = _0.93, SD = 3.43). Participants overestimated how many containers they could drink: control mean = 0.09, SD = 1.02; intervention groups overestimated even more (all *P* < 0.007), worst-performing was food label serving (mean = 1.10, SD = 1.27). Participants judged the alcohol content of beers more accurately than wine or spirits. The inclusion of a health warning had no statistically significant effect on any measure. **Conclusions** Labels with enhanced pictorial representations of alcohol content improved knowledge and understanding of the UK's low-risk drinking guidelines compared with industry-standard labels; health warnings did not improve knowledge or understanding of low-risk drinking guidelines. Designs that improved knowledge most had the low-risk drinking guidelines in a separate statement located beneath the graphics.
Hall et al. 2020 [19]	To examine reactions to graphic versus text-only warnings for cigarettes, SSBs, and alcohol	Convenience sample N=1,360. Randomly assigned participants to view a: 1) text-only warning without efficacy information; 2) text-only warning with efficacy information, 3) graphic warning without efficacy information, or 4) graphic warning with efficacy information.	Graphic warnings outperformed text-only warnings on key predictors of behavior despite causing more reactance. Found that graphic warnings for cigarettes, SSBs, and alcohol were perceived as more effective than text-only warnings, although the magnitude of the effect was small. This finding suggests that graphic warnings may be more effective at actual behavior. Across product type, graphic warnings also led to greater fear, more thinking about the harms of consuming the product, and lower product appeal.

References

1. Godfrey SS, Laughery KR, Young SL, Vaubel KP, Brelsford JW, Laughery KA, et al. The new alcohol warning labels - how noticeable are they. In:

PROCEEDINGS OF THE HUMAN FACTORS SOCIETY : 35TH ANNUAL MEETING, VOLS 1 AND 2. SANTA MONICA: HUMAN FACTORS AND ERGONOMICS SOC; 1991. p. 446–50.

2. Gorn GJ, Lavack AM, Pollack CR, Weinberg CB. An experiment in designing effective warning labels. Health Mark Q. 1996;14(2):43–61.

3. Al-hamdani M, Smith S. Alcohol warning label perceptions: Emerging evidence for alcohol policy. Can J Public Health [Internet]. 2015;106(6):e395–400. Available from: https://www.embase.com/search/results?subaction=viewrecord&id=L610692371&from=export

4. Monk RL, Westwood J, Heim D, Qureshi AW. The effect of pictorial content on attention levels and alcohol-related beliefs: An eye-tracking study. J Appl Soc Psychol [Internet]. 2017;47(3):158–64. Available from: https://www.scopus.com/inward/record.uri?eid=2-s2.0-85016220291&doi=10.1111%2Fjasp.12432&partnerID=40&md5=8ac518157c539d7700c9b74d8b082d3a

5. Wigg S, Stafford LD. Health warnings on alcoholic beverages: Perceptions of the health risks and intentions towards alcohol consumption. PLoS One [Internet]. 2016;11(4). Available from: https://www.embase.com/search/results?subaction=viewrecord&id=L611092834&from=export

6. Zahra D, Monk RL, Corder E. “IF You Drink Alcohol, THEN You Will Get Cancer”: Investigating How Reasoning Accuracy Is Affected by Pictorially Presented Graphic Alcohol Warnings. Alcohol Alcohol. 2015 Sep;50(5):608–16.

7. Annunziata A, Pomarici E, Vecchio R, Mariani A. Nutritional information and health warnings on wine labels: Exploring consumer interest and preferences. Appetite [Internet]. 2016;106:58–69. Available from: https://www.scopus.com/inward/record.uri?eid=2-s2.0-84960153061&doi=10.1016%2Fj.appet.2016.02.152&partnerID=40&md5=ad72bca8f80ed24fc2927b84faf0ff95

8. Annunziata A, Pomarici E, Vecchio R, Mariani A. Health warnings on wine: a consumer perspective. Br Food J [Internet]. 2016;118(3):647–59. Available from: https://www.scopus.com/inward/record.uri?eid=2-s2.0-84959418823&doi=10.1108%2FBFJ-08-2015-0300&partnerID=40&md5=4302a131c112b35b140b0c06b17233f4

9. Annunziata A, Pomarici E, Vecchio R, Mariani A. Do Consumers Want More Nutritional and Health Information on Wine Labels? Insights from the EU and USA. Nutrients [Internet]. 2016 Jul 7;8(7):416. Available from: https://www.ncbi.nlm.nih.gov/pubmed/27399767

10. Coomber K, Jones SC, Martino F, Miller PG. Predictors of awareness of standard drink labelling and drinking guidelines to reduce negative health effects among Australian drinkers. Drug Alcohol Rev. 2017 Mar;36(2):200–9.

11. Pham C, Rundle-Thiele S, Parkinson J, Li S. Alcohol warning label awareness and attention: A multi-method study. Alcohol Alcohol [Internet]. 2018;53(1):39–45. Available from: https://www.embase.com/search/results?subaction=viewrecord&id=L620226681&from=export

12. Al-Hamdani M, Smith SM. Alcohol Warning Label Perceptions: Do Warning Sizes and Plain Packaging Matter? J Stud Alcohol Drugs [Internet]. 2017;78(1):79–87. Available from: https://www.embase.com/search/results?subaction=viewrecord&id=L618374717&from=export

13. Dossou G, Gallopel-Morvan K, Diouf J-F. The effectiveness of current French health warnings displayed on alcohol advertisements and alcoholic beverages. Eur J Public Health [Internet]. 2017;27(4):699–704. Available from: https://www.embase.com/search/results?subaction=viewrecord&id=L622713568&from=export

14. Authayarat W, Kasornsuwan P, Jiamsanguanwong A. Design and validation of affective warning pictorials on alcohol container labels [Internet]. Vol. 585, Advances in Intelligent Systems and Computing. 2018. p. 34–42. Available from: https://www.scopus.com/inward/record.uri?eid=2-s2.0-85021752388&doi=10.1007%2F978-3-319-60495-4_4&partnerID=40&md5=6e7b03777ee4052985b16e2dc401171d

15. Sillero-Rejon C, Attwood AS, Blackwell AKM, Ibáñez-Zapata J-A, Munafò MR, Maynard OM. Alcohol pictorial health warning labels: the impact of self-affirmation and health warning severity. BMC Public Health [Internet]. 2018;18(1):1403. Available from: https://www.embase.com/search/results?subaction=viewrecord&id=L625628908&from=export

16. Vasiljevic M, Couturier D-L, Frings D, Moss AC, Albery IP, Marteau TM. Impact of lower strength alcohol labeling on consumption: A randomized controlled trial. Heal Psychol [Internet]. 2018;37(7):658–67. Available from: https://www.embase.com/search/results?subaction=viewrecord&id=L621857599&from=export

17. Annunziata A, Agnoli L, Vecchio R, Charters S, Mariani A. Health warnings on wine labels: a discrete choice analysis of Italian and French Generation Y consumers. Wine Econ Policy [Internet]. 2019;8(1):81–90. Available from: https://www.scopus.com/inward/record.uri?eid=2-s2.0-85064747618&doi=10.1016%2Fj.wep.2019.03.001&partnerID=40&md5=cff4c0c26800c9d5bbd83f7abe3bea94

18. Gold N, Egan M, Londakova K, Mottershaw A, Harper H, Burton R, et al. Effect of alcohol label designs with different pictorial representations of alcohol content and health warnings on knowledge and understanding of Low Risk Drinking Guidelines: A randomized controlled trial. Addiction [Internet]. 2020; Available from: https://www.embase.com/search/results?subaction=viewrecord&id=L633393591&from=export

19. Hall MG, Grummon AH, Maynard OM, Kameny MR, Jenson D, Popkin BM. Causal Language in Health Warning Labels and US Adults’ Perception: A Randomized Experiment. Am J Public Health [Internet]. 2019;109(10):1429–33. Available from: https://www.embase.com/search/results?subaction=viewrecord&id=L629094677&from=export
