# Supplementary material for: Alcohol Health Warning Labels: A Rapid Review with Action Recommendations
Source: Int J Environ Res Public Health. 2022 Sep 16;19(18):11676. doi: 10.3390/ijerph191811676 (PMC9517222; doi:10.3390/ijerph191811676)
Supplement: Supplementary file 1 [file ijerph-19-11676-s001.zip › S. Table S10 Alcohol industry revised Aug. 24.pdf]

Supplementary Table S10: Alcohol Industry and Alcohol Warning Labels

Author & Year	Location & Goal	Design & source of data	Impacts/outcome
Fox et al. 1998 [1]	Augusta, Georgia. Eye-tracking to monitor how subjects view print ads	Viewed ads on screen and forwarded to the next one when they wanted to. N= 143 high school students age 14-18	Beer ad, the voluntary message encouraging responsible drinking had limited warning value. However, results indicate that the disclosure used in the Miller Lite ad is less effective at attracting and holding adolescents' attention than currently mandated cigarette warnings, which have not been effective among adolescents
Matthews et al. 2013 [2]	Australia. Strategies and arguments used by segments of the alcohol industry to delay the introduction of mandatory health warning labels in Australia	Document Analysis and submissions to Australian government re FASD. 2013	Industry questioned the rationale and evidence base for labels; arguing that they will cause damage to public health and the economy; lobbying and seeking to influence government and political representatives including through monetary donations; and introducing its own voluntary labelling scheme. The arguments made against the introduction of mandatory health warning labels for alcohol are flawed and their empirical basis is limited.
Coomber et al. 2015 [3]	Australia. Evaluate awareness of the 'Get the facts' logo and alcohol warning labels, and to also evaluate consumer use of the DrinkWise website.	Online research panel N=561 age 18-45	Participants who engaged in more frequent binge drinking, those who consumed alcohol directly from a can or bottle, and participants who supported the use of health warning labels were all significantly more likely to be aware of alcohol warning labels. Older participants were significantly less likely than 18–24 year olds be aware of any of the warning labels. Older drinkers and females were less likely to be aware of the warning labels and visit the website, respectively.
Petticrew et al. 2016a [4]	England. In the United Kingdom, alcohol warning labels are the	Survey of alcohol labelling data. Analysis of UKs	Product labels assessed in relation to: information on alcohol units, government consumption guidelines, pregnancy warnings, reference to the Drinkaware website and a responsibility statement. Assessed the size, colour and placement of text, and the size and colouring of the pregnancy warning logo. The first three (required)

Supplementary Table S10: Alcohol Industry and Alcohol Warning Labels

	subject of a voluntary agreement between industry and government. Industry pledged that 80 % of products would have WLs	100 top selling brands (n=156 individual products). 2014.	elements were present on 77.6% of products examined. The mean font size of the Chief Medical Officer's (CMO) unit guidelines (usually on the back of the product) was 8.17-point. The mean size of pregnancy logos was 5.95mm. The pregnancy logo was on average smaller on wine containers.
Jones et al. 2017 [5]	Melbourne. How alcohol industry developed responsible drinking campaigns are perceived and interpreted by the general public. What is the perceived clarity of these messages compared to a government-developed campaign.	Study One (an intercept survey) age 16 and older n=180. Study two (an online survey) social network n=177 and commercial recruitment n=310.	Diversity in participants' interpretation of the `responsible drinking' advertisements. Terminology utilised in industry-developed advertisements was found to be ambiguous. In Study Two, the government-developed campaign (know when to say when) was clearly interpreted as warning against harmful consumption of alcohol. However, both of the SAPRO-developed and industry-developed adult-targeted campaigns (Drinkwise, Australia and Budweiser, US respectively) were interpreted to have a range of different meanings± including some seemingly unrelated to alcohol
Coomber et al. 2018 [6]	Victoria, Australia. To understand young adult drinkers' perceptions of current voluntary Australian alcohol product warnings. Drinkwise	Six focus groups N=40 University students, age 18-25,	Most commented on the small size of the warning relative to overall product labels. Commented on typical placement on lower back reduced visibility. Overall quality was poor. Most said they could not relate to the warning messages or that they would be relevant to their group. Even sign about pregnancy and alcohol did not send clear message.
Tinawi et al. 2018 [7]	New Zealand. Prevalence and design elements of the voluntary	Purpose sample of 59 local and imported	A majority (80%) of the alcoholic beverage containers had a pregnancy-related warning, 73% had industry-led initiatives (e.g. advising 'responsible' consumption) and 19% had drink driving/heavy machinery warnings. Warning labels were small,

	health warning labels and related industry initiatives	beers and wines. 2016-2017.	with the average area of pregnancy-related and drink-driving/heavy machinery pictograms being 45 and 36 mms, respectively (i.e. pea-size).
Farace et al. 2020 [8]	Southern Italy. To assess the visibility and effectiveness of responsible consumption messages used for the sale of the product "beer" (on packaging and in advertisements).	Three focus groups. Teenagers, age 16–17 years, and two young adult panels, age 20–24 years.	The weak efficacy of the "drink responsibly" communication campaigns carried out by beer manufacturers. Most interviewees failed to remember the existence of the "drink responsibly" messages and, even after supplementary visual stimulation, they were mostly disinterested
Vallance et al. 2020 [9]	Yukon, Ireland. Examine the framing of news coverage of alcohol warning label (AWL) initiatives that included a cancer message on alcohol containers in two different countries. Policy contexts and industry perspectives were also evaluated	Identified and systematically reviewed news articles published between 2017–2019 covering an AWL academic study in Yukon, Canada. 2017-2019.	News coverage of AWLs with a cancer message was more supportive in Canada than Ireland, where alcohol industry perspectives were consistently forwarded. Industry arguments opposing the cancer label bore similarities across contexts, often distorting or denying the evidence. Increasing awareness of industry messaging strategies may generate more critical coverage of industry lobbying activities and increase public support for alcohol policies.
Gleeson, O'Brien 2021 [10]	Melbourne. To map the new instruments containing these rules of the Tran-Pacific Agreement (TPP) and examine developments in the rules with	Trade agreements signed after the TPP negotiations concluded were retrieved and searched for alcohol-	The best legal interpretation of the TPP supplementary labelling rules indicates that they do not impede the right of governments to mandate warnings and other health information on alcoholic beverages, including the content of the messages, the inclusion of graphics, their colours and font, and their size and position on the container. The general public health exception and the A-HKFTA specific alcohol exception provide further legal support for governments wishing to mandate labelling requirements which might prevent or limit industry control over label content and placement

	implications for health information on alcohol containers standard label	specific labelling provisions. A legal analysis of these provisions and related exceptions was undertaken. Examined 88 documents. 2015	

References

1. Fox RJ, Krugman DM, Fletcher JE, Fischer PM. Adolescents' attention to beer and cigarette print ads and associated product warnings. J Advert [Internet]. 1998;27(3):57–68. Available from: https://www.scopus.com/inward/record.uri?eid=2-s2.0-0002365876&doi=10.1080%2F00913367.1998.10673563&partnerID=40&md5=676be81918776806c86827a63a2c725b

2. Mathews R, Thorn M, Giorgi C. Vested interests in addiction research and policy. Is the alcohol industry delaying government action on alcohol health warning labels in Australia? Addiction [Internet]. 2013;108(11):1889–96. Available from: https://www.embase.com/search/results?subaction=viewrecord&id=L603386636&from=export

3. Coomber K, Martino F, Barbour IR, Mayshak R, Miller PG. Do consumers "Get the facts"? A survey of alcohol warning label recognition in Australia. BMC Public Health. 2015 Aug;15:816.

4. Petticrew M, Douglas N, Knai C, Durand MA, Eastmure E, Mays N. Health information on alcoholic beverage containers: has the alcohol industry's pledge in England to improve labelling been met? Addiction [Internet]. 2016;111(1):51–5. Available from: https://www.embase.com/search/results?subaction=viewrecord&id=L616572507&from=export

5. Jones SC, Hall S, Kypri K. Should I drink responsibly, safely or properly? Confusing messages about reducing alcohol-related harm. PLoS One [Internet]. 2017 Sep 21;12(9):e0184705–e0184705. Available from: https://www.ncbi.nlm.nih.gov/pubmed/28934269

6. Coomber K, Hayley A, Miller PG. Unconvincing and ineffective: Young adult responses to current Australian alcohol product warnings. Aust J Psychol [Internet]. 2018;70(2):131–8. Available from: https://www.scopus.com/inward/record.uri?eid=2-s2.0-85028748191&doi=10.1111%2Fajpy.12177&partnerID=40&md5=b7f5919c59079a536ae87c1fa425b60a

7. Tinawi G, Gray T, Knight T, Glass C, Domanski N, Wilson N, et al. Highly deficient alcohol health warning labels in a high-income country with a voluntary system. Drug Alcohol Rev [Internet]. 2018;37(5):616–26. Available from:

https://www.embase.com/search/results?subaction=viewrecord&id=L624966104&from=export

8. Farace B, Apicella A, Tarabella A. The sustainability in alcohol consumption: the “drink responsibly” frontier. Br Food J [Internet]. 2020;122(5):1593–610. Available from: https://www.scopus.com/inward/record.uri?eid=2-s2.0-85082941070&doi=10.1108%2FBFJ-07-2019-0563&partnerID=40&md5=683775ab9ecc86326f2c2f9e60ea1064

9. Vallance K, Vincent A, Schoueri-Mychasiw N, Stockwell T, Hammond D, Greenfield TK, et al. News Media and the Influence of the Alcohol Industry: An Analysis of Media Coverage of Alcohol Warning Labels With a Cancer Message in Canada and Ireland. J Stud Alcohol Drugs [Internet]. 2020;81(2):273–83. Available from: https://www.embase.com/search/results?subaction=viewrecord&id=L631802674&from=export

10. Gleeson D, O’Brien P. Alcohol labelling rules in free trade agreements: Advancing the industry’s interests at the expense of the public’s health. Drug Alcohol Rev. 2021 Jan;40(1):31–40.
