# Supplementary material for: Alcohol Health Warning Labels: A Rapid Review with Action Recommendations
Source: Int J Environ Res Public Health. 2022 Sep 16;19(18):11676. doi: 10.3390/ijerph191811676 (PMC9517222; doi:10.3390/ijerph191811676)
Supplement: Supplementary file 1 [file ijerph-19-11676-s001.zip › S. Table S11 Alcohol policy. revised Aug. 24. docx.pdf]

Supplementary Table S11. Alcohol policy analysis and AWLs

| **Author & Year** | **Location** | **Goal** | **Design & source of data** | **Impacts/outcome** |
|---|---|---|---|---|
| Lemmens et al. 1999 [1] | US, 1985-1991 | US. The study assessed the portrayal of alcohol-related issues in print media in the US during the 7 year period bracketing the implementation of the US alcohol warning labels act in November 1988 | All articles that appeared from 1985 to 1991 in 5 national newspapers (n=1,677). Content analysis of a 15% sample of these articles. 1985-1991. | Unlike the dynamic developments in the period from 1978 through 1985, attention to alcohol-related issues in the print media did not show dramatic changes in the period from 1985 through 1991, although drunk driving remained the alcohol-related issue receiving the most national coverage |
| Wettlaufer et al. 2017 [2] | Canada, 2012 | Canada. To facilitate the dissemination of policies that reduce alcohol-related harms by documenting, comparing, and sharing information on effective alcohol polices related to restrictions on alcohol marketing and alcohol warning messaging in 10 Canadian provinces. | This analysis is part of a larger project that examined 10 policy dimensions. Compared Alcohol Marketing and Alcohol Warning Message Policies across Canada. 2017. | The national average score was 52% for restrictions on marketing policies and 18% for alcohol warning message policies. Most provinces had marketing regulations that went beyond the federal guidelines with penalties for violating marketing regulations. The provincial liquor boards' web pages focused on product promotion, and there were few restrictions on sponsorship activities. No province has implemented alcohol warning labels, and Ontario was the sole province to have legislated warning signs at all points-of-sale. Most provinces provided a variety of warning signs to be displayed voluntarily at points-of-sale; however, the quality of messages varied. |
| Kongats et al. 2020 [3] | Alberta, Quebec, 2016 | Alberta, Quebece. To understand support for population-level healthy public policies to reduce alcohol-related harm by assessing the attitudes of policy influencers and the public in two Canadian provinces. | A stratified sample of general public (n=2,400) and census sample of policy influencers (n=2,400) based on the Chronic Disease Prevention Survey. 2016 | One exception related to warning labels; overall, only 68.5% of the public and 65.4% of policy influencers supported "mandatory front of package graphic health warning labels on alcohol beverages. Among policy influncers, 64% Alberta and 68% Quebec supported mandatory warning labels on bottles |

Supplementary Table S11. Alcohol policy analysis and AWLs

**References**


1. Lemmens PH, Vaeth PAC, Greenfield TK. Coverage of beverage alcohol issues in the print media in the United States, 1985-1991. Am J Public Health. 1999 Oct;89(10):1555–60.
2. Wettlaufer A, Cukier S, Giesbrecht N. Comparing Alcohol Marketing and Alcohol Warning Message Policies Across Canada. Subst Use Misuse. 2017;52(10):1364–74.
3. Kongats K, McGetrick JA, Thomson M, Raine KD, Nykiforuk CIJ. Policy Influencer and General Public Support for Proposed Alcohol Healthy Public Policy Options in Alberta and Quebec, Canada. J Stud Alcohol Drugs [Internet]. 2020;81(1):47–57. Available from: https://www.embase.com/search/results?subaction=viewrecord&id=L630926761&from=export
