# Supplementary material for: Alcohol Health Warning Labels: A Rapid Review with Action Recommendations
Source: Int J Environ Res Public Health. 2022 Sep 16;19(18):11676. doi: 10.3390/ijerph191811676 (PMC9517222; doi:10.3390/ijerph191811676)
Supplement: Supplementary file 1 [file ijerph-19-11676-s001.zip › S. Table S2 Messages Content revised.. Aug. 24.pdf]

Supplementary Table S2. Message Content Alcohol Warning Labels

Author & Year	Location & Goal	Design & source of data	Impacts/outcome
Andrews et al. 1991 [1]	US. Assess differences in believability, favourable view and confidence in AWL between frequent and occasional or non-users	Convenience sample of 273 undergrad marketing students surveyed. Assessed labels: pregnancy and birth defects; impairment to drive a car; risk of hypertension, liver disease, cancer; dangerous in combination with other drugs; alcohol is a drug and may be addictive. Prior to Nov. 18, 1989 when US AWL implemented	Generally believability greater for occasional/nonusers than frequent users. Attitude to label is found to be significantly more favourable for occasional/nonuser vs. frequent users. Occasional/nonusers are found to be significantly more confident in their label attitudes than frequent users of alcohol
Andrews et al. 1993 [2]	Tow US universities. To determine what is viewed as favourable and unfavourable in the warnings	273 undergrad in 2 universities. Survey/ Respondents randomly assigned to assess one of 5 warning labels	While initial attitudes toward drinking may be an important element in the formation of warning label attitudes, respondents' self-generation thoughts about the warnings play a greater role in explaining their post-warning attitudes.
Annunziata et al. 2016d [3]	France, Italy, Spain, US. Health information on wine labels, consumer support, interests and preferences.	On-line survey platform 330 Italy, 185 France, 195 Spain, 306 USA	Consumers from all countries tended to assign a high utility score to "ban on alcoholic beverages to children under 18/21 years" and "do not drive after drinking". For these two warnings, the scores tended to converge, exceeding 4.1 in each country (out of 5). Significant differences ($p < 0.001$) emerge for the statement "avoid<br>drinking alcohol during pregnancy". This warning indeed was considered, on average, more useful by US and French consumers, but was less valued by Spanish and Italians. The warning "avoid drinking alcohol when you are taking medicines", a higher utility of American consumers. Similar outcomes were found for the statement "alcohol increases the risk of violence".
Kersbergen and Field, 2017 [4]	U of Liverpool. How much attention is paid to warning labels and branding on alcohol beverage containers, and how individual differences in this are associated with individual differences	Study 1 (n=60) used eye-tracking to assess how much attention alcohol consumers pay to health information and investigated correlations between attention and drinking habits. In study 2,(n=120) we experimentally manipulated motivation to reduce drinking and attention to health warnings in	Study participants paid minimal attention to warning labels (7% of viewing time).Participants who were motivated to reduce drinking paid less attention to alcohol branding and alcohol warning labels.<br>Study 2 showed that the alcohol brief intervention decreased attention to branding compared to the control condition, but it did not affect attention to warning labels.<br>The lack of attention to warning labels, even among people who actively want to cut down, suggests that there is room for improvement in the content of health warnings on alcohol packaging.

	in drinking behaviour and motivation to change it.	order to investigate the causal relationships between them.	
Hobin et al. 2018 [5]	Ontario. To test the efficacy of alcohol labels with standard drink (SD) information and Canada's Low-Risk Drinking Guidelines (LRDGs) as compared to %ABV labels on consumers' ability to estimate alcohol intake.	A between-groups experiment (n = 2,016) in which participants each viewed one of six labels. Using an online survey, participants viewed an alcohol label and were asked to estimate: (a) the amount in a SD; (b) the number of SDs in an alcohol container and (c) the number of SDs to consume to reach the recommended daily limit in Canada's LRDG.	Results indicated that labels with SD and LRDG information facilitated more accurate estimates of alcohol consumption and awareness of safer drinking limits across different beverage types (12.6% to 58.9% increase in accuracy), and labels were strongly supported among the majority (66.2%) of participants.
Sillero-Rejon et al. 2018a [6]	Spain. Examined whether enhancing self-affirmation among a population of drinkers, prior to viewing threatening alcohol pictorial health warning labels, would reduce defensive reactions and promote reactions related to behaviour change.	Expermental human laboratory study (n=128) with a control group.	There was no clear evidence that enhancing self-affirmation influenced any outcome. In comparison to moderately severe health warnings, highly-severe health warnings increased avoidance and reactance and were perceived as more effective and increased motivation to drink less
Hall et al. 2019 [7]	US. To examine US adults' reactions to	Rrecruited a convenience sample of 1,413 adults to participate in an	Most participants (76.3%) selected the warning that used "causes" as the 1 that most discouraged them from wanting to use the product. "Causes" was also

	health warnings with strong versus weak causal language	online experiment. Randomly assigned participants, to view 4 health warnings for either cigarettes, SSBs, or alcohol. Warning statements for each product used 4 causal language variants presented simultaneously in a random order: “causes,” “contributes to,”“can contribute to,” and “may contribute to.”	selected most often (39.0% of participants) as the warning that participants most supported implementing.<br>By contrast, most (66.1%) chose “may contribute to” as the warning that least discouraged them from wanting to use the product. Few demographic differences in were observred, suggesting that warnings with strong causal language are equally compelling to and supported by consumers with diverse characteristics.
Escandon-Barbosa and Rialp-Criado 2019 [8]	Columbia. To analyze the influence of the content of the product’s label on the purchase intention for the product.	N=114. Laboratory with more than 100 wines. Eye tracking and participants in front of approx.. 100 wines and possible to show 1200 observations regarding participants and the combinations of information of the information on the wine label.	**Hypothesis 1:** experts have greater effects on their purchase intention by attending to the three components of the label than do non-experts.<br>**Hypothesis 2:** Compared with women Men show a greater effect on their purchase intention by considering the three components of the label.
Annunziata et al 2019 [9]	Italy France. The influence of alternative formats of WLs on Millennial consumers stated choices of wine and beer; the existence of different segments of consumers with different level of influence of AWLs	Respondents (n=394 italy; 265 France) were subjected to two Discrete Choice Experiments (DCEs), depicting the hypothetical choice of a bottle of wine and beer.	For beer positive utility is associated with the logo warning about the risks of drinking and driving, which is actually common on the bottles of beer both in Italy and France. When it comes to choosing s bottle of beer, people prefer to be informed about the possible negative consequences of consumption, but with a neutrally framed message. When it comes to choosing wine, they prefer no warning message. Considering the warning content, consumers attached a negative utility to the brain damage logo for both beer and wine
Clarke and Rose, 2020 [10]	University of Liverpool. To assess the impact of glass labels conveying unit information and a health warning in reducing ad libitum alcohol consumption.	Experiment. 162 young adults (84 females). Mixed-methods approach: a between subjects cluster-randomized experimental study and 2 qualitative focus groups.	There was no significant main effect, indicating participants did not differ in their drinking by glass type.<br>Most noticed the unit and warning labels and did not believe that these influenced their intake. No significant main effect of condition on change in alcohol urge<br>Most participants highlighted that the information could be of use for monitoring consumption but that it was unlikely to be used to consume within the guidelines

Supplementary Table S2. Message Content Alcohol Warning Labels

Clarke et al. 2020 [11]	UK. Assess the possible impact of (i) image-and-text, (ii) text only, and (iii) image-only health warning labels [HWLs]. on selection of alcoholic versus non-alcoholic drinks.	On line experiment. Between-subjects randomised experiment with a 2 (image: present versus absent) × 2 (text: present versus absent) factorial design. Participants (n = 6024) were adults over the age of 18 who consumed beer or wine regularly (i.e. at least once a week). Interventions. Participants were randomised to one of four groups varying in the HWL displayed on the packaging of alcoholic drinks: (i) image-and-text HWL; (ii) text-only HWL; (iii) image-only HWL; and (iv) no HWL. HWLs depicted bowel cancer, breast cancer and liver cancer, which were each displayed twice across six alcoholic drinks. Each group viewed six alcoholic and six non-alcoholic drinks and selected one drink that they would like to consume	Alcohol drink selection was lower when drinks displayed a HWL. Compared to not having any label, all HWLs increased scores on each secondary outcome – negative emotional arousal, reactance, avoidance and disease risk.<br>Perceived disease risk in all three HWL groups did not show evidence of being different from each other<br>Selection of alcohol lowest for HWLs that included an image. Image-and text HWLs reduced the odds of selecting an alcoholic drink compared with text-only HWLs (OR = 0.80, 95% CI = 0.69, 0.92), but increased the odds of selecting an alcoholic drink compared with image-only HWLs (OR = 1.34, 95% CI = 1.16, 1.55)
Pechey et al. 2020 [12]	UK. To describe the potential effectiveness and acceptability of image-and-text (also known as pictorial or graphic) HWLs applied to: i. alcoholic drinks and ii. energy-dense snack foods.	Two on line studies using between subjects design. N=5,528	For both alcoholic drinks and energy-dense snacks, HWLs depicting bowel cancer generated the highest levels of negative emotional arousal and lowest desire to consume the product, but were the least acceptable. Acceptability was generally low for HWLs applied to alcohol, with 3 of 21 rated as acceptable. The majority of free-text comments expressed negative reactions to HWLs on alcohol or energy-dense snacks.

References:

1. ANDREWS JC, NETEMEYER RG, DURVASULA S. EFFECTS OF CONSUMPTION FREQUENCY ON BELIEVABILITY AND ATTITUDES TOWARD ALCOHOL WARNING LABELS. J Consum Aff. 1991;25(2):323–38.

Supplementary Table S2. Message Content Alcohol Warning Labels

2. ANDREWS JC, NETEMEYER RG, DURVASULA S. THE ROLE OF COGNITIVE RESPONSES AS MEDIATORS OF ALCOHOL WARNING LABEL EFFECTS. J PUBLIC POLICY \& Mark. 1993;12(1):57–68.

3. Annunziata A, Pomarici E, Vecchio R, Mariani A. Do Consumers Want More Nutritional and Health Information on Wine Labels? Insights from the EU and USA. Nutrients [Internet]. 2016 Jul 7;8(7):416. Available from: https://www.ncbi.nlm.nih.gov/pubmed/27399767

4. Kersbergen I, Field M. Visual attention to alcohol cues and responsible drinking statements within alcohol advertisements and public health campaigns: Relationships with drinking intentions and alcohol consumption in the laboratory. Psychol Addict Behav J Soc Psychol Addict Behav. 2017 Jun;31(4):435–46.

5. Hobin E, Vallance K, Zuo F, Stockwell T, Rosella L, Simniceanu A, et al. Testing the efficacy of alcohol labels with standard drink information and national drinking guidelines on consumers' ability to estimate alcohol consumption. Alcohol Alcohol [Internet]. 2018;53(1):3–11. Available from: https://www.embase.com/search/results?subaction=viewrecord&id=L620226676&from=export

6. Sillero-Rejon C, Attwood AS, Blackwell AKM, Ibáñez-Zapata J-A, Munafò MR, Maynard OM. Alcohol pictorial health warning labels: the impact of self-affirmation and health warning severity. BMC Public Health [Internet]. 2018;18(1):1403. Available from: https://www.embase.com/search/results?subaction=viewrecord&id=L625628908&from=export

7. Hall MG, Grummon AH, Maynard OM, Kameny MR, Jenson D, Popkin BM. Causal Language in Health Warning Labels and US Adults' Perception: A Randomized Experiment. Am J Public Health [Internet]. 2019;109(10):1429–33. Available from: https://www.embase.com/search/results?subaction=viewrecord&id=L629094677&from=export

8. Escandon-Barbosa D, Rialp-Criado J. The impact of the content of the label on the buying intention of a wine consumer. Front Psychol [Internet]. 2019;9(JAN). Available from: https://www.scopus.com/inward/record.uri?eid=2-s2.0-85059975610&doi=10.3389%2Ffpsyg.2018.02761&partnerID=40&md5=e3ad55b12df99034b922406731c5adde

9. Annunziata A, Agnoli L, Vecchio R, Charters S, Mariani A. Health warnings on wine labels: a discrete choice analysis of Italian and French Generation Y consumers. Wine Econ Policy [Internet]. 2019;8(1):81–90. Available from: https://www.scopus.com/inward/record.uri?eid=2-s2.0-85064747618&doi=10.1016%2Fj.wep.2019.03.001&partnerID=40&md5=cff4c0c26800c9d5bbd83f7abe3bea94

10. Clarke N, Rose AK. Impact of Labeled Glasses in a Bar Laboratory Setting: No Effect on Ad Libitum Alcohol Consumption. Alcohol Clin Exp Res [Internet]. 2020;44(8):1666–74. Available from: https://www.embase.com/search/results?subaction=viewrecord&id=L2005415877&from=export

11. Clarke N, Pechey E, Kosīte D, König LM, Mantzari E, Blackwell AKM, et al. Impact of Health Warning Labels on Selection and Consumption of Food and Alcohol Products: Systematic Review with Meta-analysis. Health Psychol Rev [Internet]. 2020;1–39. Available from: https://www.embase.com/search/results?subaction=viewrecord&id=L632036915&from=export

12. Pechey E, Clarke N, Mantzari E, Blackwell AKM, De-Loyde K, Morris RW, et al. Image-and-text health warning labels on alcohol and food: potential effectiveness and acceptability. BMC Public Health [Internet]. 2020;20(1):376. Available from: https://www.embase.com/search/results?subaction=viewrecord&id=L631407779&from=export
