# Supplementary material for: Alcohol Health Warning Labels: A Rapid Review with Action Recommendations
Source: Int J Environ Res Public Health. 2022 Sep 16;19(18):11676. doi: 10.3390/ijerph191811676 (PMC9517222; doi:10.3390/ijerph191811676)
Supplement: Supplementary file 1 [file ijerph-19-11676-s001.zip › S. Table S3 Perception and Awareness. revised. Aug. 24. docx.pdf]

Supplementary Table S3. Perception & Awareness of Alcohol Warning Labels

**Author & year**	**Location & Goal**	**Design & source of data**	**Impact/outcome**
Scammon et al. 1991 [1]	Utah. Evaluate the new US federal AWLs on alcoholic beverage containers	Before and after implementation of AWL. 6 surveys of rep. sample of 400 households, 2417 respondents	The labels appear to have been noticed, but there is no evidence yet that perceptions of the risks of alcohol abuse or behavior have been altered in response.
Marin, 1994 [2]	San Francisco. The self-reported awareness of product warning messages and signs among random samples of Hispanics in San Francisco	Cross sectional N=1,204	In general, respondents reported low levels of awareness of the presence of product warning messages and signs. Length of exposure to warning messages and multiplicity of sources (for example, advertisements and products) seem to produce greater levels of awareness of the presence of product warning messages. Less acculturated, Spanish-speaking Hispanics are less likely to report being aware of the warnings, particularly those that appear only in English (for example, alcoholic beverages).
Parker et al. 1994 [3]	California. To assess recall and awareness of risk	Telephone survey pre and post, 5 waves, 1989 to 1992. Pre n=913; post n=1,542	Significantly higher recall of labels and their content in the post-introduction period. both drinking drivers and impaired drivers (based on self-reports) were more likely to recall the labels and their content; an indication that warning labels are reaching "at risk" individuals<br>warning labels, as one part of a larger social movement, are helping to create an atmosphere in which drinking and driving is less acceptable.
Parsons et al. 1993 [4]	Illinois. AWL awareness and content familiarity	Survey of random sample of 481 homeless persons	Age and level of alcohol consumption were each associated with label awareness and content familiarity, suggesting that AWLs may be reaching homeless persons.
deTurck et al. 1995 [5]	US Eastern university. Hypotheized that red signal creates greater perception of danger; and green signal creates greater	Respondents received 5 different warning messages on computer monitor and then asked to recall information and indicate their	Perceived product as most dangerous when they attempted to memorize the warning and signal word was red.<br>Least dangerous when attempted to form an attitude about the alcoholic beverage and the signal word was red<br>Females rated alcoholic beverages more dangerous than males<br>Persons who consumed alcoholic beverages more frequently perceived the alcoholic beverages as much less dangerous

	perception of danger when product users are processing safety information processing objectives.	perception of product safety. 198 students (118 females)	
Marín & Gamba, 1997a [6]	San Fransico. Self-reported awareness of product warning messages.	Four independent, randomly-selected samples of Hispanic residents. (Total N 4661, 1989-1992	Hispanics in San Francisco reported varying levels of awareness of product warning messages and that there may be a moderate cumulative effect of continued exposure so that the level of awareness would tend to increase as length of exposure increases.
Marin, 1997b [7]	San Franciisco. To analyze the possible changes in awareness of AWLs and messages	Before and after n=777 Hispanics; 234 non-Hispanics, 1991, 1992	Overall, both samples showed an increase in awareness of a general product warning label for both beer and wine containers. High and low acculturated Hispanics showed an increase in awareness,
MacKinnon & Lapin, 1998 [8]	Arizona State University. Two experiments examined the effects of warnings and advertisements on memory, intentions, and benefit and risk perceptions.	164 undergrad. Students.The experiments designed to assess if AWLs may have a boomerang effect such that drinkers perceive alcohol as having more benefits when the warning is present.	From the results of Experiment 1 it was unclear whether the finding of a boomerang effect on product benefits was replicable. Experiment 2 was improved to have sufficient power to detect this effect. The boomerang effect was not observed on either the benefits or intentions dependent measures and was very close to zero. Exposure to the warning led to higher perceptions of product risk and more accurate memory for the warning. The presence of the warning counteracted the effect of the advertisement on increasing perceived benefits of drinking.
Brown et al. 2016 [9]	UK. Assess the immediate effect of alcohol warning advertisements on	Participants randomly assigned to view advertisements	Viewing alcohol warning advertisements reduces negative implicit attitudes toward alcohol in heavier drinkers, suggestive of a reactive effect.

	implicit and explicit attitudes towards alcohol and on alcohol seeking behaviour	either: alcohol promoting; alcohol warning; or unrelated to alcohol. N=373 age 18-40	
Coomber et al. 2017a [10]	Australia. investigating awareness of a number of short- and long-term consequences of alcohol use among Australian adult drinkers. It also explored demographic correlates.	Online research panel n=1,061	Fewer were aware of long-term consequences. Lastly, participants who were aware of any alcohol warning label were significantly more likely than participants not aware of warning labels to respond 'definitely true' to harm to unborn babies (OR=1.34, 95%CI=1.00-1.78, p=0.048; 70% vs. 64%) and cirrhosis of the liver (OR=1.50, 95%CI=1.13-1.99, p=0.005; 66% vs. 57%). Although only 38% of Australian drinkers report awareness of a warning re harm to unborn baby or cirrhosis of the liver.
Coomber et al. 2017b [11]	Australia. Awareness of standard drink labels and the relationship between awareness of these labels and consumer knowledge of the guidelines.	On-line research panel. Age 18-45, Final sample 1,061 participants. Data weighted by age, gender, state of residence.	Almost all participants had heard of a standard drink and 80% recognized the standard drink logo. Those who were mid-SES, who lived in a rural or regional area, and who were high-risk drinkers were significantly more likely to be aware of the logo.Participants provided a significantly higher estimate of the maximum number of standard drinks that could be consumed to reduce harm.Overall, 73% of participants supported the inclusion of information about recommended daily guidelines to reduce high-risk health effects on labels for alcohol products. Women were significantly more likely than men to support the inclusion of such information, as were those with a tertiary or higher level of education and low-risk drinkers.
Schoueri-Mychasiw et al , 2020a [12]	Northern Canada. Tested the extent to which consumers	Quasi-experimental study was conducted in two	After the intervention, unprompted and prompted recall of the drinking guideline label message increased 2.9 times more in the intervention versus comparison site. In addition, knowledge of the daily and weekly drink limits increased 1.5 and 1.4 times more in the intervention versus comparison site, respectively.

	recalled alcohol labels with national drinking guidelines and examined the impact of labels on awareness and knowledge of the guidelines.	jurisdictions in northern Canada. 2,049 cohort participants in each site	
Schoueri-Mychasiw et al, 2020b [13]	Northern Canada. The impact of an alcohol labelling intervention on recall of and support for standard drink (SD) labels, estimating the number of SDs in alcohol containers, and intended and unintended use of SD labels.	Quasi-experimental study was conducted in two jurisdictions in northern Canada. 2,049 cohort participants in each site	Participants in the intervention relative to the comparison site had greater odds of recalling and supporting SD labels and lower odds of reporting using SD labels to purchase high strength, low-cost alcohol. Exposure to the labels had negligible effects on accurately estimating the number of SDs and using SD labels to drink within guidelines.

References

1. SCAMMON DL, MAYER RN, SMITH KR. ALCOHOL WARNINGS - HOW DO YOU KNOW WHEN YOU HAVE HAD ONE TOO MANY. J PUBLIC POLICY \& Mark. 1991;10(1):214–28.

2. Marín G. Self-reported awareness of the presence of product warning messages and signs by Hispanics in San Francisco. Public Health Rep. 1994;109(2):275–83.

3. PARKER RN, SALTZ RF, HENNESSY M. THE IMPACT OF ALCOHOL BEVERAGE CONTAINER WARNING LABELS ON ALCOHOL-IMPAIRED DRIVERS, DRINKING DRIVERS AND THE GENERAL-POPULATION IN NORTHERN CALIFORNIA. ADDICTION. 1994 Dec;89(12):1639–51.

4. Parsons JA, Johnson TP, Barrett ME. Awareness and knowledge of alcohol beverage warning labels among homeless persons in cook county, illinois. Int Q Community Health Educ. 1993 Jan;14(2):153–64.

Supplementary Table S3. Perception & Awareness of Alcohol Warning Labels

5. deTurck MA, Goldhaber GM, Richetto GM. Effectiveness of alcohol beverage warning labels: Effects of consumer information processing objectives and color of signal word. J Prod Toxics Liabil [Internet]. 1995;17(3):187–95. Available from: https://www.scopus.com/inward/record.uri?eid=2-s2.0-0342742684&partnerID=40&md5=96fe229a73b422d4afc8f7541236769e

6. Marín G, Gamba RJ. Changes in reported awareness of product warning labels and messages in cohorts of California Hispanics and non-Hispanic whites. Heal Educ Behav Off Publ Soc Public Heal Educ. 1997 Apr;24(2):230–44.

7. Marin G. Changes across 3 years in self-reported awareness of product warning messages in a Hispanic community. Health Educ Res. 1997 Mar;12(1):103–16.

8. MacKinnon DP, Lapin A. Effects of alcohol warnings and advertisements: A test of the boomerang hypothesis. Psychol \& Mark. 1998 Oct;15(7):707–26.

9. Brown KG, Stautz K, Hollands GJ, Winpenny EM, Marteau TM. The cognitive and behavioural impact of alcohol promoting and alcoholwarning advertisements: An experimental study. Alcohol Alcohol [Internet]. 2016;51(3):354–62. Available from: https://www.embase.com/search/results?subaction=viewrecord&id=L610272649&from=export

10. Coomber K, Jones SC, Martino F, Miller PG. Predictors of awareness of standard drink labelling and drinking guidelines to reduce negative health effects among Australian drinkers. Drug Alcohol Rev. 2017 Mar;36(2):200–9.

11. Coomber K, Mayshak R, Curtis A, Miller PG. Awareness and correlates of short-term and long-term consequences of alcohol use among Australian drinkers. Aust N Z J Public Health [Internet]. 2017;41(3):237–42. Available from: https://www.embase.com/search/results?subaction=viewrecord&id=L619837720&from=export

12. Schoueri-Mychasiw N, Weerasinghe A, Stockwell T, Vallance K, Hammond D, Greenfield TK, et al. Use as directed: do standard drink labels on alcohol containers help consumers drink (ir)responsibly? Real-world evidence from a quasi-experimental study in Yukon, Canada. Drug Alcohol Rev. 2021 Feb;40(2):247–57.

13. Schoueri-Mychasiw N, Weerasinghe A, Vallance K, Stockwell T, Zhao J, Hammond D, et al. Examining the Impact of Alcohol Labels on Awareness and Knowledge of National Drinking Guidelines: A Real-World Study in Yukon, Canada. J Stud Alcohol Drugs [Internet]. 2020;81(2):262–72. Available from: https://www.embase.com/search/results?subaction=viewrecord&id=L631801263&from=export
