# Supplementary material for: Alcohol Health Warning Labels: A Rapid Review with Action Recommendations
Source: Int J Environ Res Public Health. 2022 Sep 16;19(18):11676. doi: 10.3390/ijerph191811676 (PMC9517222; doi:10.3390/ijerph191811676)
Supplement: Supplementary file 1 [file ijerph-19-11676-s001.zip › S. Table S5 College students. revised Aug 24. docx.pdf]

Supplementary Table S5. Young adults, college students and alcohol warning labels

Author & year	Location & Goal	Design & source of data	Impact/outcome
Andrews et al, 1990 [1]	US. (1) to determine the believability of and attitudes toward AWLs, (2) to determine if prior attitudes and beliefs toward drinking alcohol mediate the influence of believability and attitudes toward the labels and (3) to examine whether attitudes toward alcohol label information are influenced by attitudes toward drinking alcohol	Quasi-experimental . 273 undergrad marketing students from two universities, 1989	While all warnings are rated as believable, the ones regarding birth defects and driving impairment are perceived to be significantly more believable than the others. In addition, persons with more favourable attitudes toward alcohol consumption tend to disbelieve specific instance hazards (e.g., birth defects, driving impairment and drug combination warnings), while disliking long-term risks of alcohol consumption and abuse (e.g., hypertension, liver disease, cancer and addiction warnings).
Snyder and Blood, 1992 [2]	US. Conducted an experiment to test the effects of the newly introduced Surgeon General's alcohol warnings and advertisements	40 college students assigned to 4 experimental conditions (n=159)	The warnings boomeranged, causing drinkers to perceive greater benefits from the alcoholic beverages. The advertisements had powerful effects on both drinkers and nondrinkers, increasing perceptions of benefits and decreasing risks. The results suggest that, for young people,<br>the warning labels in their present form may be counterproductive, and advertisements make drinking more attractive.
MacKinnon and Fenaughty, 1993 [3]	Arizona State University. This article reports the relationship between substance use and memory for health warnings for cigarettes, smokeless tobacco, and alcohol.	Measures of substance use and recognition memory for warning labels were collected from 2 samples of college students (n = 288 and n = 243).	It appears that substance users process the content of the warning label to an extent that it improves their memory for the risks on the label. The results suggest that heavier users, the group at higher risk for the problems described on the warning label, are also most likely to retain the content of the warning.
Malouff et al, 1993 [4]	US. To determine(1) how conspicuous the federally required alcohol warning is, (2)	Four studies of male and female	In all four studies few of the drinkers could recall the main parts of the warnings, even though the warning had been required on all alcohol containers for over two years. In Study 1, 77% thought that the warning was not conspicuous but could be made conspicuous through various

	what might make it more conspicuous, (3) how well recalled the warning is, (4) whether the warning, if delivered in a way that made it more conspicuous would be better recalled and (5) whether an alcohol warning with enhanced saliency would lead to less drinking	college students Overall n=154.	changes, such as by printing the warning horizontally (parallel to the brand name) rather than vertically. In Study 2, respondents rated the horizontal warning significantly more conspicuous. In Study 3, subjects with the horizontal warning later showed significantly better awareness/recall of the warning .In Study 4, the half of the subjects prompted to notice the alcohol warning drank less thereafter in the bar than the other subjects.
Garretson and Burton 1994 [5]	Southern US<br>1. Examine whether attitudes about alcohol, warnings, differ for those below and above legal drinking age<br>2. Assess if perceptions of warning message impacted by the sponsor of the message<br>3. Assess if attitudes toward promotional item influenced by brand information	2 brand info x 3 warning info x 2 consumer over and under legal age.Company sponsored warning, government warning and no-warning control condition	• Those of legal drinking age hold more favourable attitudes toward alcohol and perceive less risk associated with consumption, compared to those below legal age<br>• Awareness of risks of driving after drinking and drinking while pregnant generally where high across all warning conditions<br>• When beverage marketer sponsors the message, the social concern of the marketer is perceived as more positive than when US govt or no warning is provided<br>• More favourable attitude toward sales promotion item when it includes brand characters than when no brand characters are included.
DeCarlo et al, 1997 [6]	US. Large southeastern university. Test several hypotheses H1: A direct positive relationship exists between individuals' perceived health consciousness and the perceived effectiveness of alcohol warnings and warning labels.<br>H2: There is an inverse relationship between rates of alcohol consumption and the favorability of perceptions of	Asked to review several alcohol warnings and warning labels and rate on effectiveness, Overall 150 undergrad University	H1 and H2 not supported. Moreover, consumption of alcohol was negatively related to the behavior of reading product warning labels. Thus, while individuals who drink more alcohol do not perceive warnings and warning labels to be less effective, they may selectively reduce their exposure to such messages.<br>H3. No significant differences by age in perception of effectiveness of AWL<br>H4. Received some support, as the two messages which were both theoretically ranked as the most intense and<br>also estimated to embody the greatest source credibility (the ACDA's "More Americans have died on the highways…" and the Surgeon General's "Women should not drink during pregnancy…") were rated as the most potentially effective.<br>Those who view themselves as health conscious, a greater willingness to ready warnings and labels. Some support for social judgement theory

	alcohol warning<br>and warning label effectiveness.<br>H3: Older consumers will perceive alcohol warnings and warning labels to be more effective than will younger consumers<br>H4: There is a direct positive relationship between perceived message effectiveness and the language intensity of a high-credibility source.		
Creyer et al, 2002<br>[7]	Australia & US. This research examines how two different alcoholic beverage health warnings placed on the label of a fictitious brand<br>of beer influence alcohol-related risk perceptions, attitudes and intentions,and characterizations of problem-drinking behaviors of binge and non-binge drinkers in Australia and the United States	Subjects were randomly assigned to experimental conditions and data were collected in a classroom setting. N=274 undergrad from two universities	Findings offer mixed results for the effect of the type of warning (“Alcohol is a Drug” versus the standard warning used in the U.S.). The warning type had no significant effect on social or health benefits or other attitudinal variables, but it had diverse effects on the perceived risk measures and perceptions of various drinking behaviors. For binge drinkers, use of the standard U.S. warning resulted in lower risk perceptions than the “Alcohol is a Drug” warning/ After being exposed to the “Alcohol is a Drug” warning, binge drinkers’ perceptions of the drinking patterns are more strongly impacted than perceptions of non-bingers.
Jones and Gregory, 2009<br>[8]	New South Wales. To examine young people’s perceptions of standard drink labelling, the purposes for which they would use standard drink information, and the potential impact on their alcohol consumption levels	A total of six focus groups, six and 10 per group, were conducted with students enrolled in a university course in a large regional city in New South Wales.	The focus group participants had a reasonable knowledge of standard drinks and high awareness of standard drink labelling. However, it was clear was that they used this information not to reduce their alcohol consumption, and thus their exposure to potential alcohol related harms, but rather to increase or even maximise their alcohol consumption. Even more alarming is the participants’ perceptions—based on their personal experiences— that this harm-maximisation use of standard drink labelling is even more prevalent among underage drinkers
Glock and Krolak-	University of Saarland; Luxembourg. This study	Half of the participants	Results imply a greater effectiveness of the warning labels that contradicted positive alcohol outcome expectancies compared

Supplementary Table S5. Young adults, college students and alcohol warning labels

Schwerdt. 2013 [9]	compared the effectiveness of warning labels that contradicted positive outcome expectancies with health-related WLs	received health-related warning labels while half received positive related warning labels. Implicit attitudes were assessed before and after warning-label exposure. N=40.	to the warning labels that illustrated the health-damaging consequences of alcohol consumption. First, although the two groups differed in their implicit attitudes at baseline, positive-related and health-related warning labels differentially influenced the implicit attitudes of the two groups. In the health-related warning labels group, implicit attitudes turned into more positive ones, whereas in the positive-related warning labels group, implicit attitudes tended to become more negative after exposure to warning labels
Jarvis and Pettigrew, 2013 [10]	Australia. University. The first research objective was to provide an empirical assessment of whether warning statements can dissuade young consumers from purchasing in the presence of information relating to brand and alcohol content. The second research objective was to observe the patterns of these preferences across a number of different warning statements.	Sample from web panel provider, age 18-21 and 22-25	In general, negatively framed health statements had greater impact on at-risk groups, which were defined as those with higher reported alcohol consumption. The negatively framed messages impacted three of the five classes overall. By comparison, the positively framed statement relating to driving had a positive influence on choice, indicating that such a message may actually increase the probability of purchasing products bearing this statement. Results showed that warning messages can influence choice in the presence of other attributes.
Krischler and Glock, 2015 [11]	Luxembourg. Germany. We formulated the warnings as questions or as statements and expected that the warning labels that were posed as questions but not the warning labels that were presented as statements would be able to change outcome expectancies and drinking intentions compared with a control group.	60 undergrad from U. of Luxembourg; 50 from Justus Liebig U. Giessen, Germany, 2013, 2014	The results of this experiment revealed that warning labels presented as statements had no influence on individual and general outcome expectancies and drinking intentions. Warnings posed in the form of questions showed some effectiveness as they increased individual negative outcome expectancy perception.

Supplementary Table S5. Young adults, college students and alcohol warning labels

Armitage and Arden, 2016 [12]	UK. University. Two studies were designed to test the hypotheses that (a) alcohol warning labels augmented with a self-affirming implementation intention would significantly decrease subsequent alcohol consumption, and (b) any effect of self-affirmation on alcohol consumption would be mediated by greater message acceptance and increased motivation	Two experiments. Recruited from university campuses via advertisements on screensavers and staff e-newsletters. Paid 5 Sterling. Randomly assigned to one of two conditions. Self-affirming implementation intention and; effect of self-affirming implementation intention.	In two studies, we showed that engaging in a wine-pouring task and being exposed to a self-affirming implementation intention led to significant decreases in alcohol consumption at follow-up. Given the brevity of the intervention and the multiple domains in which it might be deployed (e.g., cigarette packaging), the present findings are encouraging and warrant further investigation.<br>Two studies showed that standard information augmented with a self-affirming implementation intention was capable of significantly reducing alcohol consumption. Although it is not yet clear which variables mediate the observed effects, the present research demonstrates potential for deploying a simple intervention with considerable public health “reach” in reducing alcohol consumption specifically and health behaviors more generally.
Annunziata et al, 2017 [13]	University of Naples. Are Italian university students interested in receiving warnings on alcoholic beverage labels? Are there distinct segments of university students with different attitudes towards warning labels and different preferences?	Quantitative survey of a sample of university students, on line survey, 2016	Almost half the interviewees (48%) paid sporadic attention to the information on the labels of alcoholic drinks and 28% never read the labels on alcoholic beverages.The warning logo “do not drive after drinking” has the highest visibility (27% of respondents claimed they had noticed it often). In terms of the exposure effect, half the sample stated that the information did not have any influence on their behaviour; while 19% revealed they avoided driving after drinking and 16% said they had reflected on the potential risks of drinking and driving. Considering the five specific warnings selected from the Eurocare library, on average, respondents found the following recommendations more useful: do not drink while taking medicine and do not drink and drive. The results also confirm the evidence provided by previous research that individuals with a higher alcohol consumption frequency are least interested in health warnings on labels.
Jones et al, 2021 [14]	Glasgow. To explore, in depth, young adult drinkers’ awareness of the health information and messaging currently displayed on alcohol packaging, how it is perceived	Focus groups were conducted in Glasgow (Scotland) in September 2019 with 50 young	Unaided recall of some health information and messaging was high (e.g. units, pregnancy symbols); however, most participants did not attend to or meaningfully engage with these, viewing them as unnoticeable, obscure and ineffective.Participants were skeptical of alcohol companies’ motivations with respect to health messaging on products. They were surprised to see the novel warnings on alcohol products but generally supported their inclusion. In this study, those who supported introducing warnings maintained they should be noticeable, fact-

	and if it is used, and also perceptions of novel health warnings.	adult past-month drinkers segmented by age (18–24, 25–35), gender (female, male), and social grade (ABC1, C2DE)	based and relevant to real life. Most thought that these warnings could increase awareness of alcohol-related harms, particularly for younger or potential drinkers. Large, combined (text and image) warnings with specific messages on the front of packaging were considered most engaging and potentially effective.<br>Most groups thought that the cancer warning would be more believable, informative and effective if the types of cancers caused by alcohol consumption were specified, which could help to improve awareness and knowledge of the alcohol cancers link. Some participants contended that alcohol companies would not want salient health-related information or warnings on alcohol packaging, viewing it as detrimental to sales and positive perceptions of drinking. This was evident in participants' attitudes to responsible drinking messages, considered ambiguous and positively-framed, and the fact that other health-related messaging and information is seen as barely noticeable.

References

1. ANDREWS JC, NETEMEYER RG, DURVASULA S. BELIEVABILITY AND ATTITUDES TOWARD ALCOHOL WARNING LABEL INFORMATION - THE ROLE OF PERSUASIVE COMMUNICATIONS THEORY. J PUBLIC POLICY \& Mark. 1990;9:1–15.

2. Snyder LB, Blood DJ. Caution: Alcohol Advertising and the Surgeon General's Alcohol Warnings May Have Adverse Effects on Young Adults. J Appl Commun Res [Internet]. 1992;20(1):37–53. Available from: https://www.scopus.com/inward/record.uri?eid=2-s2.0-84933489182&doi=10.1080%2F00909889209365318&partnerID=40&md5=baf90451f51330d6a755a809846fddbf

3. MacKinnon DP, Fenaughty AM. Substance use and memory for health warning labels. Health Psychol [Internet]. 1993;12(2):147–50. Available from: https://www.embase.com/search/results?subaction=viewrecord&id=L23899063&from=export

4. MALOUFF J, SCHUTTE N, WIENER K, BRANCAZIO C, FISH D. IMPORTANT CHARACTERISTICS OF WARNING DISPLAYS ON ALCOHOL CONTAINERS. J Stud Alcohol. 1993 Jul;54(4):457–61.

5. Garretson JA, Burton S. Alcoholic beverage sales promotion: An initial investigation of the role of warning messages and brand characters among consumers over and under the legal drinking age. J PUBLIC POLICY \& Mark. 1998;17(1):35–47.

6. DeCarlo TE, Parrott R, Rody R, Winsor RD. Alcohol warnings and warning labels: An examination of alternative alcohol warning messages and perceived effectiveness. J Consum Mark [Internet]. 1997;14(6):448–60. Available from: https://www.scopus.com/inward/record.uri?eid=2-s2.0-0345880034&doi=10.1108%2F07363769710186060&partnerID=40&md5=4675faf4af28864bcf4341f3bf0f59ac

7.. Creyer EH, Kozup JC, Burton S. An experimental assessment of the effects of two alcoholic beverage health warnings across countries and binge-drinking status. J Consum Aff. 2002;36(2):171–202.

8.. Jones SC, Gregory P. The impact of more visible standard drink labelling on youth alcohol consumption: Helping young people drink (ir)responsibly? Drug

Alcohol Rev. 2009 May;28(3):230–4.

9.. Glock S, Krolak-Schwerdt S. Changing Outcome Expectancies, Drinking Intentions, and Implicit Attitudes toward Alcohol: A Comparison of Positive Expectancy-Related and Health-Related Alcohol Warning Labels. Appl Psychol WELL BEING. 2013 Nov;5(3):332–47.

10. Jarvis W, Pettigrew S. The relative influence of alcohol warning statement type on young drinkers' stated choices. FOOD Qual Prefer. 2013 Apr;28(1):244–52.

11. Krischler M, Glock S. Alcohol warning labels formulated as questions change alcohol-related outcome expectancies: A pilot study. Addict Res Theory [Internet]. 2015;23(4):343–9. Available from: https://www.scopus.com/inward/record.uri?eid=2-s2.0-84944071307&doi=10.3109%2F16066359.2015.1009829&partnerID=40&md5=31d6bac584abb2bc9f3642857f6918aa

12. Armitage CJ, Arden MA. Enhancing the effectiveness of alcohol warning labels with a self-affirming implementation intention. Heal Psychol [Internet]. 2016;35(10):1159–63. Available from: https://www.embase.com/search/results?subaction=viewrecord&id=L610736403&from=export

13. Annunziata A, Vecchio R, Mariani A. Alcohol warnings and moderate drinking patterns among Italian university students: An exploratory study. Nutrients [Internet]. 2017;9(6). Available from: https://www.embase.com/search/results?subaction=viewrecord&id=L616824219&from=export

14. Jones D, Moodie C, Purves RI, Fitzgerald N, Crockett R. Health information, messaging and warnings on alcohol packaging: a focus group study with young adult drinkers in Scotland. Addict Res \& THEORY. 2021;
