# Supplementary material for: Alcohol Health Warning Labels: A Rapid Review with Action Recommendations
Source: Int J Environ Res Public Health. 2022 Sep 16;19(18):11676. doi: 10.3390/ijerph191811676 (PMC9517222; doi:10.3390/ijerph191811676)
Supplement: Supplementary file 1 [file ijerph-19-11676-s001.zip › S. Table S6 Adults and General Population. revised..Aug. 24.pdf]

S. Table S6: Adults and General Population and Alcohol Warning Labels

Author & Year	Location & Goal	Design & source of data	Impacts/outcomes
Kaskutas and Greenfield, 1991 [1]	US. This study addresses who has seen these labels and respondents' reported changes in relevant knowledge, attitudes and behavior	Surveys of 2, 006 adults and 2,000 adults. June, July 1989; June, July, August 1990	Greater proportion of key target groups had seen AWLs. Those who saw them more likely to report heightened awareness of hazards of drinking.Young male drivers more llkely to have seen AWLs.The warning labels on alcoholic beverage containers were most widely seen by the heavy drinkers in the overall sample and by the heavy drinkers in each of the target groups. The finding that those seeing the warning label also were more likely to report having conversations about the hazards of drinking during pregnancy reinforces the notion that the labels serve as a reminder of already-known hazards
Graves, 1993 [2]	US and Ontario, Canada. To evaluate the impact of the introduction of a health warning label on alcoholic beverage containers.	National household telephone surveys conducted in the US (2,000) in 1989, 1990, and 1991; and in the Province of Ontario, Canada (1,000) in 1990 and 1991.	27% of US respondents vs 16% in Ontario reported having seen WL. in 1990<br>35% in US and 19% in Ontario reported seeing WL in 1991.Of those having seen it 7.5 significantly more in US than in Ontario recall seeing birth defecs. Recall of drinking and driving message declined over one year [1990 to 1991]. Compared to US respondents, Ontario respondents less likely to perceive driving after drinking as dangerous.Respondents who saw label more likely to talking about alcohol and pregnancy and drinking and driving. Higher penetration of AWL awareness among, heavy drinkers, young men, and young women
Greenfield and Kaskutas, 1993 [3]	US. This paper reports on the first and second in a series of three annual waves to assess the effects of AWLs in the general U.S. population.	Surveys of rep. samples of adults; 2,006 in July 1989 and June-August in 1990	Six months after introduction of warning labels, over one fifth of the respondents reported having seen the labels. Greater proportions of key target groups, such as young men at risk for drunk driving and heavy drinkers, reported seeing the warnings. Strength of belief in the truth of included label content increased significantly but very slightly. The findings suggest that the current warning labels are being noticed by many of those at risk of hazards discussed in the labels. Health information on alternative messages was initially less well known and knowledge levels declined, suggesting these also be considered as suitable warning messages. Over one fourth (27%) of the women of childbearing age also saw the warning labels compared to 11% for all others , with the heavy drinkers in this group, again, the most likely to have seen the labels (almost 40%) as compared to the "other" drinkers (28%).

S. Table S6: Adults and General Population and Alcohol Warning Labels

Kaskutas. Greenfield 1992 [4]	US. This study addresses who has seen these labels and respondents' reported changes in relevant knowledge, attitudes and behavior.	National survey s pre and post AWL introduction. 1989 n=2,006; 1990 n=2,000	Greater proportions of key target groups, such as heavy drinkers and young men at risk for drunk driving, reported seeing the warnings. After controlling for demographics and alcohol consumption, respondents who probably saw the warning labels were significantly more likely to report several behaviors indicative of heightened awareness of, and caution regarding, the hazard of drinking and driving and of drinking during pregnancy, hazards that are both included on the warning labels. Among those targeted by the warning label messages, young male drivers (deemed at risk for drunk driving) were among the more frequent respondents to have seen the warning label on alcohol beverage containers:<br>Over one fourth of the women of childbearing age also saw the warning labels compared to a tenth for all others, with the heavy drinkers in this group, again, the most likely to have seen the labels as compared to the 'other' drinkers. The finding that those seeing the warning label also were more likely to report having conversations about the hazards of drinking during pregnancy reinforces the notion that the labels serve as a reminder of already-known hazards. The warning labels on alcoholic beverage containers were most widely seen by the heavy drinkers in the overall sample and by the heavy drinkers in each of the target groups.
Kaskutas, 1993 [5]	US. To assess the effects of AWLs.	National surveys 1989 n=2,006 1991 n=2,017	18 months after introduction level of support for the warning label mandate was significantly higher than that found prior to its introduction. In 1991, 91% of the respondents answered that they favored the AWL. Having been exposed to the warning label also is found to increase the likelihood of support for the policy; respondents assessed as having seen the warning label were more than twice as likely (as those who did not see the label) to respond in favor of the label (OR = 2.20). In addition, survey year is found to be predictive of support; respondents in 1991 were almost one and one quarter times as likely to be in favour of the warning label policy (OR = 1.23). Men are found in this logistic analysis to be only half as likely as women to support the policy (OR = .55). Persons who reported seeing the label on a beverage container were twice as likely as others to support the warning label policy. More than 1/2 of the respondents believed warning labels were an effective way to change people's behaviour.
Greenfield et al, 1999 [6]	US & Ontario, Canada.Whether early trends re AWLs are maintained in the longer term.	National surveys 1990 n=2,006; 1991 n= 2,017; 1993 n=1,026; 1994 n=1,016	In the U.S., penetration peaked in 1993–94, with 43% of the lifetime drinkers reporting label awareness. Those seeing labels in the U.S. were more likely to engage in conversations about drinking and driving than those not seeing. Reports of limiting drinking for health reasons showed a positive association with label exposure increasing with time. Findings from this quasi-experiment cannot establish causal relationships, but the pattern of results, though mixed, suggests modest effects on conversations and several precautionary behaviors related to risks of drinking.
Kozup et al. 2001 [7]	US. How do drinkers' and non-drinkers' attitudes towards the wine product and perceptions of the long-term	A 2 (health-effect claim: no claim vs. health-effect claim) × 2 (wine drinking status: drinker vs.	The consumer's wine drinking status will influence (a) attitude towards the product, (b) the perceived role of wine in a healthy diet, and (c) inferred long-term health consequences of wine?. **Supported**<br>Drinking status will moderate consumers' responses to (a) a warning statement and (b) a health claim presented on an alcoholic beverage container. Specifically, a warning statement will have a positive influence (a boomerang effect) on drinkers' (but not on nondrinkers') attitudes, perceptions, and inferences. Conversely, a health claim will have a positive influence on nondrinkers (but not on drinkers') attitudes, perceptions, and inferences. **Not supported**

	health benefits associated with alcohol consumption differ.? How do the availability of a health effects claim, a warning statement, and the consumer's drinking status interact to influence consumers' attitudes towards the product and perceptions of the perceived long-term health effects of wine consumption?	nondrinker) × 2 (government warning statement: no warning vs. warning). N=150 mailed questionnaire	Consistent with the boomerang effect, our results indicate that drinkers of wine had more favorable attitudes towards the product and perceptions of health-related consequences when a warning was available compared to when it was not. Furthermore, the availability of a health claim was sufficient to induce a boomerang effect in response to the warning among nondrinkers. Thus, our findings suggest that a health-effects claim may be an effective promotional tool to increase penetration among nondrinkers.
Tam and Greenfield, 2010 [8]	US. Hypothesized that warnings would also influence the likelihood of intervening to deter others' driving after drinking.	National surveys, before and after mandated AWLs in US N=1,376, 1988 & 1989	The predicted relationships were found between message recall and actions to deter another's drinking driving. An important preventive effect of the alcohol warning label may be to legitimate collateral attempts to avert another's drunk driving.
Jongenelis et al. 2018 [9]	Australia. Whether, among adults drinking at levels associated with long-term harm, exposure to alcohol warning	Online survey of Austrlians age 18-65. N=364	For all conditions except liver damage, the extent to which alcohol was believed to be a risk factor for a specific chronic disease was significantly greater after respondents were exposed to a statement presenting information advising of such risk. The effect sizes associated with these pre- to post-exposure changes were large, especially for the statement Alcohol increases your risk of diabetes, followed by Alcohol increases your risk of mental illness and Alcohol increases your risk of heart disease. Overall intentions to reduce consumption changed favourably pre- to post-exposure for all statements except Alcohol increases your risk of liver damage. Exposure to the Alcohol increases your risk of diabetes statement was associated with the greatest effect size. Warning statements advising of the specific chronic

	statements relating to specific chronic diseases (i) increases the extent to which alcohol is believed to be a risk factor for those chronic diseases and (ii) influences alcohol consumption intentions.		diseases associated with alcohol consumption can produce favourable changes in drinking intentions among at-risk drinkers.
Hobin et al, 2020 [10]	Whitehorse, Yukon. To examine the effects of strengthening alcohol labels on consumer attention and message processing, and a self-reported reduction in drinking due to the labels, as well as investigate whether consumer attention to and processing of the labels mediate the relationship between exposure to strengthened	Quasi-experimental study. 2017. N=2,049 unique cohort.	Generalized Estimating Equations with difference-in-difference terms were used to examine the impact of the label intervention on<br>changes in outcomes. Strengthening health messages on alcohol container labels significantly increased consumer attention to [Adjusted Odds Ratio (AOR)=17.2, 95%CI:8.2,36.2] and processing of labels (e.g., reading labels: AOR=2.6, 95%CI:1.8,3.7), and consumer reports of drinking less due to the labels (AOR=3.7, 95%CI: 2.0,7.0). Reading labels closely increased in contrast with the comparison site: Thinking about labels increased to a greater extent than comparison site;<br>Talking with others also increased to a greater extent; The percentage of participants reporting drinking less alcohol due to the labels increased to a greater extent overall between Waves 1 and 3 in the intervention versus comparison site.<br>The findings in this study strongly suggest that the way in which we communicate with drinkers about the health risks of alcohol is consequential; that is, strengthening health messages on alcohol container labels appears to change the way in which drinkers attend to, process, and behaviourally respond to the information on the labels. Generally, the results show statistically significant increases across the intervention period in all five key indicators among those exposed versus unexposed to the alcohol label intervention

	alcohol labels and a self-reported reduction in drinking.		
Hobin et al. 2020 [11]	Whitehorse, Yukon & Yellowknife, Northwest Territories. To test whether alcohol labels with a cancer warning and national drinking guidelines are an effective tool for supporting more informed and safer alcohol consumption among drinkers.	Quasi-experimental study.2017-2018. Cohort n=2,049	Rates of noticing the labels were high at baseline and follow-up in both the intervention (baseline = 80.4%, follow-up = 76.7%) and comparison (baseline = 87.0%, follow-up = 78.5%) sites. Recall of the cancer warning label increased to a greater extent in the intervention versus the comparison site. Self-reported influence to cut down on drinking (+ 4.0%vs− 0.5%, AOR = 2.5, 95% CI = 1.3, 4.7) (Fig. 2f) and reports of drinking less because of the labels (+ 3.7%vs. − 3.3%, AOR = 2.4, 95% CI = 1.3, 4.3) (Fig. 2g) increased to greater extents in the intervention versus the comparison site. Females more likely to cut back drinking than males.
Hobin et al. 2020 [12]	Whitehorse, Yukon & Yellowknife, Northwest Territories This study is the first real-world study to test if cancer warning labels on alcohol containers are an effective tool for increasing population awareness that alcohol can cause cancer. More	Quasi-experimental study. May 2017, & May 2018. Cohort n=1,647	Two months after the cancer label, unprompted (+24.2% vs. +0.6%; adjusted odds ratio [AOR] = 32.7, 95% CI [5.4, 197.7]) and prompted (+35.7% vs. +4.1%; AOR = 6.2, 95% CI [3.6, 10.9]) recall increased to a greater extent in the intervention versus com¬parison site. There was a 10% greater increase in knowledge (+12.1% vs. +11.6%; AOR = 1.1, 95% CI [0.7, 1.5]) 2 months after the cancer label in the intervention versus comparison site. Similar results were found 6 months after the cancer label for all three outcomes.<br>Unprompted recall of the cancer warning message increased to a greater extent between Wave 1 (before the cancer warning label) and Wave 2 (2 months after the can¬cer warning label was stopped) in the intervention versus comparison site (+24.2% vs. 0.6%; adjusted odds ratio [AOR] = 32.7, 95% CI [5.4, 197.7]), and between Wave 1 and Wave 3 (6 months after the cancer warning label was stopped) (+12.6% vs. +1.6%; AOR = 8.8, 95% CI [1.6, 49.4].)<br>The results indicated that those who recalled the cancer message had 2.3 greater odds of knowing alcohol can cause cancer (AOR = 2.3, 95% CI [1.9, 2.7]). The results also indicated that those who know alcohol can cause cancer are 1.6 times more likely to support health warning labels relative to those who do not know, adjusting for sociodemographics and other covariates (AOR = 1.6, 95% CI [1.38, 1.89]).

	specifically, this study tested the initial and continued effects of cancer warning labels on drinkers' recall of the cancer warning and knowledge that alcohol can cause cancer. In addition, this study describes support for health warning labels on alcohol containers and assesses the association between knowledge and support for labels.		
Roderique-Davies et al. 2020 [13]	University of South Wales. tudy one aimed to examine what shoppers attend to when purchasing alcohol, whilst study two investigated the design and placement of health messages on alcohol labels.	For study one, an eye-tracker device was used to measure gaze times in a mock shopping task. Participants were organized into two groups: low-risk signs (n = 5) and combined-risk signs (n = 20). For study two,	In conclusion, alcohol purchasing appears to be guided by brand and price predominantly. Despite positive attitudes to health warnings, participants paid little attention to them. As such, the health messages had limited influence on purchasing and harm minimization.

		the design of alcohol labels was discussed in three focus groups (n = 10).	
Zhao et al. 2020 [14]	Yukon and Northwest Territories. New evidence informed AWLs were introduced in the sole government-run liquor store in Whitehorse, Yukon, that included a cancer warning (Ca), low-risk drinking guidelines (LRDGs) and standard drink (SD) messages. These temporarily replaced previous pregnancy warning labels. We test if the intervention was associated with reduced alcohol consumption	An interrupted time series study was designed to evaluate the effects of the AWLs on consumption for 28 months before and 14 months after starting the intervention. Neighboring regions of Yukon and Northwest Territories served as control sites, 2017 – 2018.	Total per capita retail alcohol sales in Whitehorse decreased by 6.31% during the intervention. Per capita sales of labeled products decreased by 6.59%, whereas sales of unlabeled products increased by 6.91%. There was a still larger reduction occurring after the intervention when pregnancy warning labels were reintroduced (-9.97% and -10.29%, t test p < .001).

References

1. Kaskutas L, Greenfield T. Knowledge of warning labels on alcoholic beverage containers. In: Proceedings of the Human Factors Society [Internet]. Alcohol

Research Group, Berkeley, CA, United States; 1991. p. 441–5. Available from: https://www.scopus.com/inward/record.uri?eid=2-s2.0-0025846580&doi=10.1518%2F107118191786754888&partnerID=40&md5=a26ad6701fe7147822448e725ce6008c

2. GRAVES KL. AN EVALUATION OF THE ALCOHOL WARNING LABEL - A COMPARISON OF THE UNITED-STATES AND ONTARIO, CANADA IN 1990 AND 1991. J PUBLIC POLICY \& Mark. 1993;12(1):19–29.

3. Greenfield TK, Kaskutas LA. Early impacts of alcoholic beverage warning labels: National study findings relevant to drinking and driving behavior. Saf Sci [Internet]. 1993;16(5–6):689–707. Available from: https://www.embase.com/search/results?subaction=viewrecord&id=L23258344&from=export

4. Kaskutas L, Greenfield TK. First effects of warning labels on alcoholic beverage containers. Drug Alcohol Depend [Internet]. 1992;31(1):1–14. Available from: https://www.embase.com/search/results?subaction=viewrecord&id=L22320403&from=export

5. Kaskutas LA. Differential perceptions of alcohol policy effectiveness. J Public Health Policy [Internet]. 1993;14(4):413–36. Available from: https://www.embase.com/search/results?subaction=viewrecord&id=L24048349&from=export

6. Greenfield TK, Graves KL, Kaskutas LA. Long-term effects of alcohol warning labels: Findings from a comparison of the United States and Ontario, Canada. Psychol \& Mark. 1999 May;16(3):261–82.

7. Kozup J, Burton S, Greyer E. A comparison of drinkers' and nondrinkers' responses to Health-related information presented on wine beverage labels. J Consum Policy [Internet]. 2001;24(2):209–30. Available from: https://www.scopus.com/inward/record.uri?eid=2-s2.0-11144267396&doi=10.1023%2FA%3A1012277421936&partnerID=40&md5=41415ee53514bc98d97ccbba110fb1ce

8. Tam TW, Greenfield TK. Do Alcohol Warning Labels Influence Men's and Women's Attempts to Deter Others from Driving When Intoxicated? Hum FACTORS Ergon Manuf \& Serv Ind. 2010;20(6, SI):538–46.

9. Jongenelis MI, Pratt IS, Slevin T, Chikritzhs T, Liang W, Pettigrew S. The effect of chronic disease warning statements on alcohol-related health beliefs and consumption intentions among at-risk drinkers. Health Educ Res [Internet]. 2018 Oct 1;33(5):351–60. Available from: https://www.ncbi.nlm.nih.gov/pubmed/30085037

10. Hobin E, Weerasinghe A, Vallance K, Hammond D, McGavock J, Greenfield TK, et al. Testing Alcohol Labels as a Tool to Communicate Cancer Risk to Drinkers: A Real-World Quasi-Experimental Study. J Stud Alcohol Drugs [Internet]. 2020;81(2):249–61. Available from: https://www.embase.com/search/results?subaction=viewrecord&id=L631802089&from=export

11. Hobin E, Schoueri-Mychasiw N, Weerasinghe A, Vallance K, Hammond D, Greenfield TK, et al. Effects of strengthening alcohol labels on attention, message processing, and perceived effectiveness: A quasi-experimental study in Yukon, Canada. Int J Drug Policy [Internet]. 2020;77. Available from: https://www.embase.com/search/results?subaction=viewrecord&id=L2005182720&from=export

12. Hobin E, Shokar S, Vallance K, Hammond D, McGavock J, Greenfield TK, et al. Communicating risks to drinkers: testing alcohol labels with a cancer warning and national drinking guidelines in Canada. Can J Public Health [Internet]. 2020;111(5):716–25. Available from: https://www.embase.com/search/results?subaction=viewrecord&id=L631912049&from=export

13. Roderique-Davies G, Davies N, Stone B, Jones S, Leeworthy S, John B. Investigating the impact of changing health messages on alcohol products. J Subst Use [Internet]. 2020;25(6):598–604. Available from: https://www.scopus.com/inward/record.uri?eid=2-s2.0-

85083560657&doi=10.1080%2F14659891.2020.1749948&partnerID=40&md5=f79501d1e412f0a9eaeee8583cd5fd0a

14. Zhao J, Stockwell T, Vallance K, Hobin E. The Effects of Alcohol Warning Labels on Population Alcohol Consumption: An Interrupted Time Series Analysis of Alcohol Sales in Yukon, Canada. J Stud Alcohol Drugs [Internet]. 2020;81(2):225–37. Available from: https://www.embase.com/search/results?subaction=viewrecord&id=L631802708&from=export
