# Supplementary material for: Alcohol Health Warning Labels: A Rapid Review with Action Recommendations
Source: Int J Environ Res Public Health. 2022 Sep 16;19(18):11676. doi: 10.3390/ijerph191811676 (PMC9517222; doi:10.3390/ijerph191811676)
Supplement: Supplementary file 1 [file ijerph-19-11676-s001.zip › S. Table S8 Cancer Aug. 24. docx.pdf]

Supplementary Table S8: Cancer and Alcohol Warning Labels

Author & Year	Location & Goal	Design & Source of Data	Impacts/outcomes
Pettigrew et al. 2014 [1]	Australia. This study investigated the acceptability among drinkers of cancer warning statements for alcoholic beverages.	Mixed methods. Six focus groups. 11 cancer warnings. Tested on 2,168 drinkers via an online survey. 2014.	Overall, responses to the cancer statements were neutral to favorable, indicating that they are unlikely to encounter high levels of negative reaction from the community if introduced on alcoholic beverages. Females, younger respondents, and those with higher levels of education generally found the statements to be more believable, convincing, and personally relevant. Positively framed messages, those referring to specific forms of cancer, and those using 'increases risk of cancer' performed better than negatively framed messages, those referring to cancer in general, and those using the term 'can cause cancer'.
Miller et al. 2016 [2]	Australia. This study aimed to canvass responses of the Australian public to cancer warning labels on alcohol products.	National survey, n=1,600. 2014.	Overall, the labels were well received, with the majority (>70 %) agreeing all labels could raise awareness and prompt conversations about the cancer risk associated with alcohol. Around 50 % or less agreed that the labels could influence drinking behaviour, but larger proportions agreed that the labels would prompt them to discuss the issue with family and friends. Multivariate analyses demonstrated that being inclined to act upon warning abel recommendations in general was the most important predictor of agreement with all of the impact statements.
Pettigrew et al. 2016 [3]	Australia. This study investigated the potential effectiveness of alcohol warning statements designed to increase awareness of the alcohol–cancer link.	National online survey n=1,680. 2016.	Of the six statements tested, Alcohol increases your risk of bowel cancer produced the highest scores across all outcome measures. All statements produced favourable changes in alcohol consumption intentions, including among high-risk drinkers.The results of the present study indicate that cancer warning statements have the potential to encourage drinkers to reduce their alcohol consumption.
Blackwell et al. 2018	UK. Examine the influence of unit labels and	Online between-subjects	Participants reported higher motivation to drink less after viewing both cancer messages and negatively framed messages.

[4]	health warnings on drinkers' understanding, attitudes and behavioural intentions regarding drinking and examine optimal methods of delivering this information on labels.	experiment. Age 18+ n=1,884. 2018.	
Clarke et al, 2020 [5]	UK. Obtain a preliminary assessment of the possible impact of (i) image-and-text, (ii) text only, and (iii) image-only health warning labels on selection of alcoholic versus non-alcoholic drinks	A between subject design randomized experiment with a 2 (image present versus abscent) x 2 (text present vs absent) factorial design. On-line survey 6,024 respondents	It was concluded that health warning labels communicating the increased risk of cancers associated with alcohol consumption reduced selection of alcoholic versus non-alcoholic drinks. Labels displaying images had the largest effect
May et al. 2021a [6]	Australia. To gain an understanding of how the Australian public might make sense of information about alcohol-related cancer	Qualitative study, seven focus groups, total n=38	Participants expressed a negative response to the alcohol warning labels, and their talk worked to challenge the legitimacy of alcohol-related cancer messages, and the entities responsible for disseminating the information.These responses functioned to counter any implied recommendation for reduction in speakers' alcohol consumption.

	risk, and how this knowledge might be used to make decisions about personal behaviour.		
Ma 2021 [7]	Australia. This study examined the role of pictorial warning labels (PWLs) featuring narrative content in communicating alcohol-related cancer risks.	169 adult consumers were randomly assigned to two narrative pictorial warning labels (PWLs), two non-narrative PWLs, or control	Results showed that exposure to narrative PWLs significantly increased participants' worry about, feelings of risk of, and perceived severity of harm of getting alcohol-related cancer, but did not affect their comparative likelihood of getting alcohol-related cancer or intentions to reduce alcohol use.<br>Exposure to narrative PWLs also indirectly influenced intentions through increased worry. Moreover, participants' risk perceptions and intentions in non-narrative PWLs condition did not differ from those in narrative PWLs and control conditions.Therefore, these findings suggest that narrative PWLs are a promising strategy in informing consumers about the cancer risks of alcohol.
Vallance et al. 2020 [8]	Yukon. To assessed consumers' baseline knowledge of alcohol-related cancer risk, standard drinks, and low-risk drinking guidelines as well as levels of support for AWLs.	Baseline survey (n=836) was conducted with systematically selected liquor store patrons in the single government-run liquor store in the capital city of Whitehorse, Yukon. 2017	Overall, there was low knowledge of alcohol-related cancer risk (24.5%), limited ability to calculate a standard drink (29.5%), and low knowledge of daily (49.5%) and weekly (48.2%) low-risk drinking guideline limits. There was moderate support for AWLs with a health warning (55.9%) and standard drink information (51.4%), and lower support for low-risk drinking guideline labels (38.7%).

References

1. Pettigrew S, Jongenelis M, Chikritzhs T, Slevin T, Pratt IS, Glance D, et al. Developing cancer warning statements for alcoholic beverages. BMC Public

Health [Internet]. 2014;14:786. Available from: https://www.embase.com/search/results?subaction=viewrecord&id=L605430521&from=export

2. Miller ER, Ramsey IJ, Baratiny GY, Olver IN. Message on a bottle: Are alcohol warning labels about cancer appropriate? Health behavior, health promotion and society. BMC Public Health [Internet]. 2016;16(1). Available from: https://www.scopus.com/inward/record.uri?eid=2-s2.0-84957952773&doi=10.1186%2Fs12889-016-2812-8&partnerID=40&md5=d085e3b889b454e1a8441d002fe9a15a

3. Pettigrew S, Jongenelis MI, Glance D, Chikritzhs T, Pratt IS, Slevin T, et al. The effect of cancer warning statements on alcohol consumption intentions. Health Educ Res [Internet]. 2016;31(1):60–9. Available from: https://www.embase.com/search/results?subaction=viewrecord&id=L614557563&from=export

4. Blackwell AKM, Drax K, Attwood AS, Munafò MR, Maynard OM. Informing drinkers: Can current UK alcohol labels be improved? Drug Alcohol Depend [Internet]. 2018;192:163–70. Available from: https://www.embase.com/search/results?subaction=viewrecord&id=L2001129732&from=export

5. Clarke, N. Pechey,E. E., ,Blackwell,AKM, De-loyde,K., Morris, RW, Munafo, MR, Marteau TM, Hollands, GJ. Impact of health warning labels communicating the risk of cancancer on alcohol selection. Addiction 116: 41-52. **https://doi.org/10.1111/add.15072**s

6. May NJ, Eliott J, Crabb S. “Alcohol causes cancer”: a difficult message for Australians to swallow. Health Promot Int [Internet]. 2021; Available from: https://www.embase.com/search/results?subaction=viewrecord&id=L634502559&from=export

7. Ma, Z. (2021). The Role of Narrative Pictorial Warning Labels in Communicating Alcohol-Related Cancer Risks. *HEALTH COMMUNICATION*. https://doi.org/10.1080/10410236.2021.1888456

8.. Vallance K, Vincent A, Schoueri-Mychasiw N, Stockwell T, Hammond D, Greenfield TK, et al. News Media and the Influence of the Alcohol Industry: An Analysis of Media Coverage of Alcohol Warning Labels With a Cancer Message in Canada and Ireland. J Stud Alcohol Drugs [Internet]. 2020;81(2):273–83. Available from: https://www.embase.com/search/results?subaction=viewrecord&id=L631802674&from=export
