# Supplementary material for: Alcohol Health Warning Labels: A Rapid Review with Action Recommendations
Source: Int J Environ Res Public Health. 2022 Sep 16;19(18):11676. doi: 10.3390/ijerph191811676 (PMC9517222; doi:10.3390/ijerph191811676)
Supplement: Supplementary file 1 [file ijerph-19-11676-s001.zip › S. table S9 Public Opinion. revised August 24.pdf]

Supplementary Table S9: Public Opinion on Alcohol Warning Labels

Author & Year	Location & Goal	Design & source of data	Impacts/outcome
Hilton, Kaskutas 1991 [1]	US. Support for AWLs policy compared to other policies.	Random sample of 2,006 adults. 1989.	About 87% approved of WLs. Support was greater among women than among men (92% *v.* 83%). A lower among those in the 30-39 year category. The less respondents drank, the more likely they were to support the program. Even among heavier drinkers, a majority support the policy. 89% agreed that 'warning labels don't have much effect as far as really heavy drinkers are concerned'.
Kaskutas 1993 [2]	US. Assess the effects of alcoholic beverage warning labels	General population telephone surveys 2,006 in 1989 and 2,017 in 1991	Support for WLs was significantly higher after introduced (87% vs. 91%). Odds ratio of 4.01 is associated with abstention. Respondents who had seen WL were likely to favour it (OR = 2.20). More than 1/2 of the respondents believed WLs were an effective way to change people's behavior.
Room et al. 1995 [3]	US & Ontario. Trends in public opinion about 11 possible alcohol policy initiatives	Probability surveys of adults, 1989, 1990, 1991.	Between 1989 and 1991 the most substantial WLs net change in attitudes was in favour of AWLs in Ontario, although it was US not Canada where AWLs were introduced after the 1989 survey
Weiss et al. 1997 [4]	Israel. Opinion on warning labels and on levels of knowledge of the risks	Survey n=3,065.	Arabs were more in favour of warning labels on alcohol containers than Jews. The initial knowledge levels among the participants were not very high.
Giesbrecht. Greenfield. 1999 [5]	US & Canada. Differences with regard to views on alcohol policy topics	Cross-sectional household surveys in Canada (11,550) and the US (4,004). 1989-1990.	In Canada 76.2% support warning labels the 3rd highest of 11 policy items. In US 88.1% supported tied for 1st with education. US had introduced warning labels between 1989 and 1990 surveys. Less support among frequent or heavy drinkers than lighter drinkers
Anglin et al. 2001 [6]	Ontario. Public opinion on policy measures and respondents' characteristics	Survey of representative sample of 1,205 adults. 1998	73% said WL were a good idea. Women and those with lower education more supportive.

Supplementary Table S9: Public Opinion on Alcohol Warning Labels

Giesbrecht et al. 2005 [7]	Ontario. This study examined the association between drinking variables and views on policy	Ontario surveys aged 18 and older 1,254 in 2000 and 1,206 in 2002	68% said yes to warning labels on alcoholic beverages<br>More support among women, married or divorced, lower educated, lower income; Less support among frequent drinkers, and those with AUDIT score.
Maharaj et al. 2018 [8]	Trinidad and Tobago. To determine support for changes in a national alcohol campaign	Cross-sectional convenience samples. 1,695 households.	Households would support: placing more prominent AWLs on products displaying alcohol content (87.2%); placing more prominent AWLs on products showing harmful effects (88.5%); increasing taxes on alcohol sales (87.7%).
Thompson et al . 2012 [9]	Australia. Drinkers' views on health in formation and AWLs	Mixed methods, cross-sectional survey (1500, qualitative study (6 focus groups). 2009.	Survey found 80% to 90% support a range of information to be included in AWLs. Labels should be integrated with other health messages such as government social advertising campaigns
Dekker et al. 2020 [10]	7 countries, Australia, Canada, China, India, New Zealand, United Kingdom, US. Assess public support for a range of recommended alcohol control initiatives across seven countries Based on Alcohol Control Policy Index (ACPI)	Web panel recruitment of 1,000 adults, per country, age 18+ quotas set to match country profiles re gender, age, and income tertiles	Assessed 14 alcohol policy intiatives. Standard drink info on packages: 72% ; pregnancy AWL, 73%; health AWL 67% Support was highest for labelling requirements, in particular pregnancy warnings (67-85%) and standard drink quantity information (63-83%). Across all policies, support was generally higher in India (80-86%) and China (57-85%), and lower in the United States (33-72%) and Canada (35-68%). Support was highest for labelling requirements, being a drinker (β=-.09, p=<.001) and drinking five or more days per week (β=-.05; p=<.001) were negatively associated with overall policy support.

References

1. Hilton ME, Kaskutas L. Public support for warning labels on alcoholic beverage containers. Br J Addict [Internet]. 1991;86(10):1323–33. Available from: https://www.embase.com/search/results?subaction=viewrecord&id=L21298210&from=export

Supplementary Table S9: Public Opinion on Alcohol Warning Labels

2. Kaskutas LA. Differential perceptions of alcohol policy effectiveness. J Public Health Policy [Internet]. 1993;14(4):413–36. Available from: https://www.embase.com/search/results?subaction=viewrecord&id=L24048349&from=export

3. Room R, Graves K, Giesbrecht N, Greenfield T. Trends in public opinion about alcohol policy initiatives in Ontario and the US 1989-91. Drug Alcohol Rev. 1995;14(1):35–47.

4. Weiss S. Israeli Arab and Jewish youth knowledge and opinion about alcohol warning labels: Pre-intervention data. ALCOHOL Alcohol. 1997;32(3):251–7.

5. Giesbrecht N, Greenfield T. Public opinions on alcohol policy issues: a comparison of American and Canadian surveys. Addiction. 1999 Apr;94(4):521–31.

6. Anglin L, Kavanagh L, Giesbrecht N. Alcohol-related policy measures in Ontario: Who supports what and to what degree? Can J Public Heal [Internet]. 2001;92(1):24–8. Available from: https://www.scopus.com/inward/record.uri?eid=2-s2.0-0035088442&doi=10.1007%2Fbf03404838&partnerID=40&md5=3da38e9e8d692816258a44e3e6956e5f

7. Giesbrecht N, Ialomiteanu A, Anglin L. Drinking patterns and perspectives on alcohol policy: results from two Ontario surveys. Alcohol Alcohol. 2005;40(2):132–9.

8. Maharaj RG, Babwah T, Motilal MS, Nunes P, Brathwaite R, Legall G, et al. The National Alcohol Survey of Households in Trinidad and Tobago (NASHTT): willingness to support changes in policy, laws and regulations. BMC Public Health [Internet]. 2018;18(1):1202. Available from: https://www.embase.com/search/results?subaction=viewrecord&id=L624649301&from=export

9. Thompson KD, Stockwell T, MacDonald S. Is there a “low-risk” drinking level for youth? The risk of acute harm as a function of quantity and frequency of drinking. Drug Alcohol Rev [Internet]. 2012;31(2):184–93. Available from: http://www.embase.com/search/results?subaction=viewrecord&from=export&id=L51703357

10. Dekker MR, Jones A, Maulik PK, Pettigrew S. Public support for alcohol control initiatives across seven countries. Int J Drug Policy [Internet]. 2020;82. Available from: https://www.embase.com/search/results?subaction=viewrecord&id=L2006137307&from=export
